# New Basal Iguanodonts from the Cedar Mountain Formation of Utah and the Evolution of Thumb-Spiked Dinosaurs

**DOI:** 10.1371/journal.pone.0014075

**Published:** 2010-11-22

**Authors:** Andrew T. McDonald, James I. Kirkland, Donald D. DeBlieux, Scott K. Madsen, Jennifer Cavin, Andrew R. C. Milner, Lukas Panzarin

**Affiliations:** 1 Department of Earth and Environmental Science, University of Pennsylvania, Philadelphia, Pennsylvania, United States of America; 2 Utah Geological Survey, Salt Lake City, Utah, United States of America; 3 John Day Fossil Beds National Monument, Kimberly, Oregon, United States of America; 4 St. George Dinosaur Discovery Site at Johnson Farm, St. George, Utah, United States of America; 5 Naturalist Illustrator, Torre di Mosto, Venice, Italy; Raymond M. Alf Museum of Paleontology, United States of America

## Abstract

**Background:**

Basal iguanodontian dinosaurs were extremely successful animals, found in great abundance and diversity almost worldwide during the Early Cretaceous. In contrast to Europe and Asia, the North American record of Early Cretaceous basal iguanodonts has until recently been limited largely to skulls and skeletons of *Tenontosaurus tilletti*.

**Methodology/Principal Findings:**

Herein we describe two new basal iguanodonts from the Yellow Cat Member of the Cedar Mountain Formation of eastern Utah, each known from a partial skull and skeleton. *Iguanacolossus fortis* gen. et sp. nov. and *Hippodraco scutodens* gen. et sp. nov. are each diagnosed by a single autapomorphy and a unique combination of characters.

**Conclusions/Significance:**

*Iguanacolossus* and *Hippodraco* add greatly to our knowledge of North American basal iguanodonts and prompt a new comprehensive phylogenetic analysis of basal iguanodont relationships. This analysis indicates that North American Early Cretaceous basal iguanodonts are more basal than their contemporaries in Europe and Asia.

## Introduction

Basal, or non-hadrosaurid, members of Iguanodontia are among the most widespread, diverse, and numerous dinosaurs in Early Cretaceous terrestrial deposits. Especially rich records are known from the Wealden beds of Europe and many formations in east-central Asia [1]–[3]. Northern Africa and Australia have produced three and possibly two taxa, respectively [4], [5]. North America used to have an Early Cretaceous basal iguanodont record comparable in diversity to those of northern Africa and Australia, with the well represented *Tenontosaurus tilletti* comprising the bulk of known material, supplemented by the lesser known “*Camptosaurus*” *depressus* and *Dakotadon lakotaensis* [6]–[9].

Recent discoveries have dramatically expanded the North American basal iguanodont assemblage. A new species of *Tenontosaurus*, *T*. *dossi* from the Twin Mountains Formation of Texas, was named by Winkler et al. [10]. Brill and Carpenter [11] recognized that a skull historically referred to the Late Jurassic *Camptosaurus dispar* actually hails from the Early Cretaceous Purgatoire Formation of Colorado and accordingly made it the holotype of the new taxon *Theiophytalia kerri*. However, the greatest bounty of new discoveries has come from the Cedar Mountain Formation of eastern Utah, including *Eolambia caroljonesa*, *Planicoxa venenica*, and *Cedrorestes crichtoni* [12]–[14]. We describe herein two partial skeletons recently discovered in the Cedar Mountain Formation, which represent two new species of basal iguanodonts: *Iguanacolossus fortis* and *Hippodraco scutodens*. These new taxa not only augment the North American basal iguanodont record, but also reveal new information on basal iguanodont phylogeny.

### Geological Context

The entirely terrestrial Cedar Mountain Formation is divided into four members: in ascending stratigraphic order, the Yellow Cat, Poison Strip, Ruby Ranch, and Mussentuchit [15]. Only a few radiometric dates have been obtained thus far from the Cedar Mountain Formation: an age of approximately 124 Ma (earliest Aptian) from the uppermost Yellow Cat Member [16], [17], an age of approximately 104.5 (late Albian) from the upper Ruby Ranch Member [18], and ages of approximately 98.2–96.7 Ma (early Cenomanian) from the Mussentuchit Member [17], [19]. Both specimens described herein were discovered in the Yellow Cat Member.

**Figure 1:**
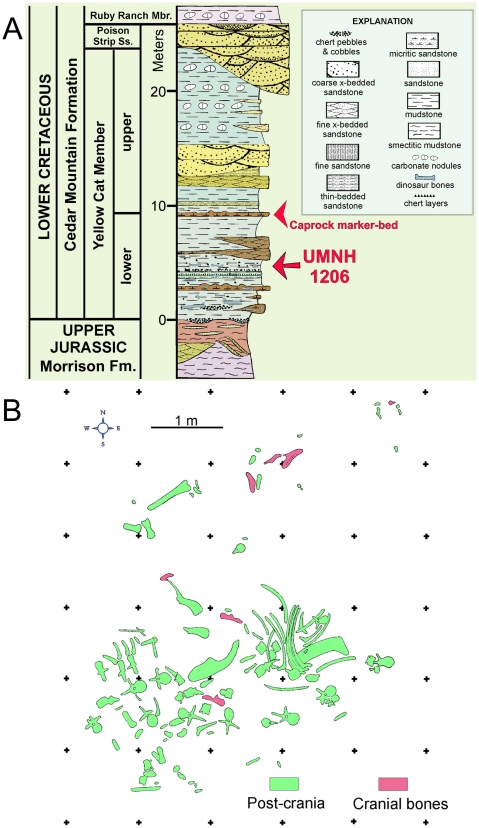
Stratigraphy and taphonomy of the type locality of *Iguanacolossus fortis*. (A) Stratigraphy of Don's Ridge, including the type locality of *Iguanacolossus fortis* (UMNH VP Locality 1206). (B) Quarry map of UMNH VP Locality 1206.

**Figure 2:**
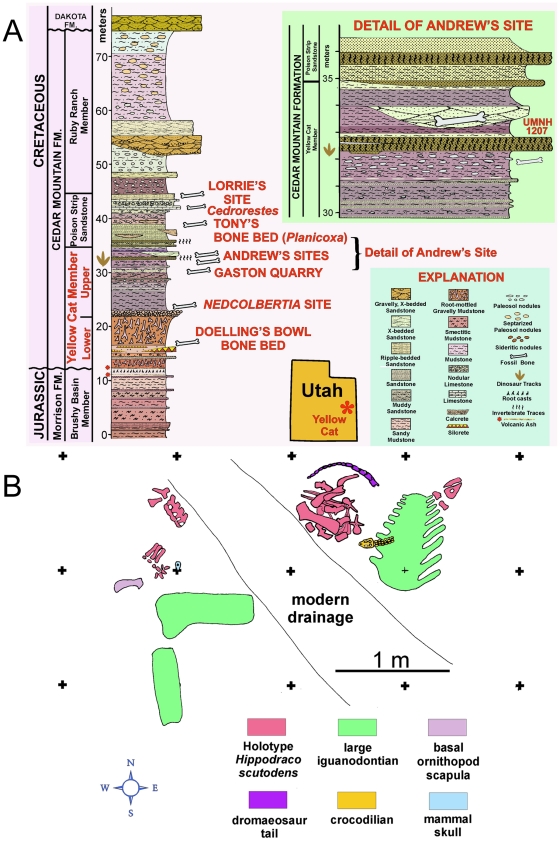
Stratigraphy and taphonomy of the type locality of *Hippodraco scutodens*. (A) Stratigraphy of Andrew's Site, including the type locality of *Hippodraco scutodens* (UMNH VP Locality 1207). (B) Quarry map of UMNH VP Locality 1207.

It has recently been recognized that the Yellow Cat Member is divisible into lower and upper portions separated by a caprock marker bed (Figs. 1, 2) [20], [21]. The holotype of *Iguanacolossus fortis*, UMNH VP 20205, was recovered below the caprock in the lower Yellow Cat at a site known as Don's Ridge (named after discoverer Don DeBlieux) (Fig. 1A). It was discovered as a single associated skeleton including disarticulated cranial and postcranial elements (Fig. 1B); the lack of element overlap and size compatibility indicate that the remains pertain to a single individual. The age of the lower Yellow Cat is difficult to ascertain, although work is underway to do so [JIK et al., in preparation]; for the purposes of this paper, it is provisionally treated as early Barremian, possibly older.

The holotype of *Hippodraco scutodens*, UMNH VP 20208, was discovered in a multispecific bonebed in a lenticular channel sandstone near the top of the Yellow Cat at a site known as Andrew's Site (named after discoverer Andrew R. C. Milner) (Fig. 2A). In addition to UMNH VP 20208, specimens recovered from this quarry include indeterminate remains of a much larger iguanodontian (see below), an indeterminate basal ornithopod (see below), an indeterminate dromaeosaurid theropod, a new crocodylomorph, and a new mammal [JIK et al., unpublished data] (Fig. 2B). A modern drainage bisected the site, eroding away portions of both UMNH VP 20208 and the larger iguanodontian. Basal iguanodont elements were assigned to the single individual UMNH VP 20208 based upon size compatibility and lack of element overlap. The age of the Andrew's Site quarry is readily determined to be late Barremian to earliest Aptian, as the aforementioned date of 124 Ma was obtained from detrital zircons sampled from mudstone and sandstone layers stratigraphically above the caprock [16; JIK, unpublished data].

Institutional Abbreviations: AMNH, American Museum of Natural History, New York, NY, USA; ANSP, Academy of Natural Sciences, Philadelphia, PA, USA; CEUM, College of Eastern Utah Prehistoric Museum, Price, UT, USA; CM, Carnegie Museum of Natural History, Pittsburgh, PA, USA; DMNH, Denver Museum of Nature and Science, Denver, CO, USA; HERM, Hull and East Riding Museum, Hull, UK; IRSNB, Institut royal des Sciences naturelles de Belgique, Brussels, Belgium; IVPP, Institute of Vertebrate Paleontology and Paleoanthropology, Beijing, China; MIWG, Museum of Isle of Wight Geology (Dinosaur Isle Museum), Sandown, UK; MNHN, Muséum national d'Histoire naturelle, Paris, France; MSM, Arizona Museum of Natural History (formerly Mesa Southwest Museum), Mesa, AZ, USA; NHMUK, Natural History Museum (formerly BMNH, British Museum of Natural History), London, UK; OXFUM, Oxford University Museum of Natural History, Oxford, UK; PIN, Palaeontological Institute, Moscow, Russia; QM, Queensland Museum, South Brisbane, Australia; SDSM, South Dakota School of Mines and Technology, Rapid City, SD, USA; SMU, Southern Methodist University Shuler Museum of Paleontology, Dallas, TX, USA; UMNH, Utah Museum of Natural History, Salt Lake City, UT, USA; USNM, National Museum of Natural History, Washington, DC, USA; YPM, Yale Peabody Museum of Natural History, New Haven, CT, USA.

## Results

### 1. Lower Yellow Cat Iguanodont

#### Systematic Paleontology

Dinosauria Owen, 1842 [22]

Ornithischia Seeley, 1887 [23]

Ornithopoda Marsh, 1881 [24]

Iguanodontia Dollo, 1888 [25] *sensu* Sereno, 2005 [26]

Styracosterna Sereno, 1986 [27] *sensu* Sereno, 2005 [26]

*Iguanacolossus fortis* gen. et sp. nov.

#### ZooBank Life Science Identifier (LSID) for genus

urn:lsid:zoobank.org:act:86F3A22B-9327-42E0-B53A-B9DD23B2CCFE.

#### ZooBank LSID for species

urn:lsid:zoobank.org:act:737FED01-0B7E-450B-8586-DF2B212CD84B.

#### Holotype

UMNH VP 20205, the associated partial skeleton of a single individual.

#### Specific Diagnosis (as for genus by monotypy)

Basal styracosternan diagnosed by a single autapomorphy: contact surface for supraoccipital on caudomedial process of squamosal is sinuous in caudal view. Also distinguished by a unique combination of characters: postorbital process of the squamosal mediolaterally compressed and blade-like; axial neural spine blade-like and semi-circular in profile; cranial extremity of preacetabular process of ilium modified into horizontal boot; dorsal margin of ilium straight; cranial pubic process with concave dorsal margin but little expansion of its cranial end (dorsal and ventral margins both curve dorsally); pubis tapers to a blunt point.

#### Locality and Horizon

Don's Ridge (discovered by DDD in 2005), UMNH VP locality 1206, near Green River, Grand County, Utah; exact locality information is on file at the Utah Geological Survey and Utah Museum of Natural History. Collected in the lower portion of the Yellow Cat Member of the Cedar Mountain Formation (? lower Barremian, Lower Cretaceous) [20], [21].

#### Etymology

*Iguanacolossus*, a combination of *Iguana* and the Latin *colossus*, in reference to the herbivorous lizards of the genus *Iguana*, the teeth of which have been historically compared to those of basal iguanodonts, and to the large size of the holotype skeleton; *fortis* from the Latin (mighty). The gender of the genus is masculine. The intended meaning of the binomen is “mighty iguana colossus”.

### Description

**Figure 3:**
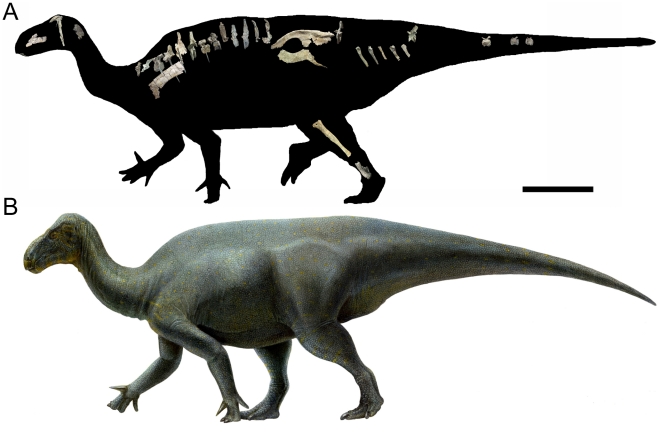
Reconstruction and restoration of *Iguanacolossus fortis*. (A) Skeletal reconstruction of *Iguanacolossus fortis*, showing the known elements of UMNH VP 20205 (the right maxilla, right squamosal, right scapula, right ilium, right pubis, and right metatarsals III and IV have been reversed for the purposes of reconstruction). (B) Life restoration of *Iguanacolossus fortis* by Lukas Panzarin. Scale bar in A equals 1 meter.

Measurements of UMNH VP 20205 are given in Table 1. *Iguanacolossus* is restored as a large, somewhat ponderous beast with robust limbs (Fig. 3) and probably of a body size similar to that of *Iguanodon bernissartensis* (∼9 meters). Cranial elements of *Iguanacolossus* recovered include a fragment of the predentary, a partial right maxilla, the right squamosal, right and left quadrates, and two loose teeth.

**Table 1:** Measurements of UMNH VP 20205, the holotype of *Iguanacolossus fortis*.

Element	Measurement
Right quadrate, dorsoventral height along caudal margin	∼32.5
Left quadrate, dorsoventral height along caudal margin	30.5
Dentary tooth, greatest preserved mesiodistal width	2.5
Maxillary tooth, greatest preserved mesiodistal width	2.3
Right ilium, preserved craniocaudal length, measured along the dorsal margin from cranial-most tip of preacetabular process to caudal-most preserved point on postacetabular process	89.5
Right pubis, craniocaudal length of prepubic process, measured along ventral margin	∼41
Right pubis, proximodistal length of postpubic process, measured along cranial margin	∼39
Left fibula, preserved proximodistal length	63.0

Measurements are given in centimeters.

**Figure 4:**
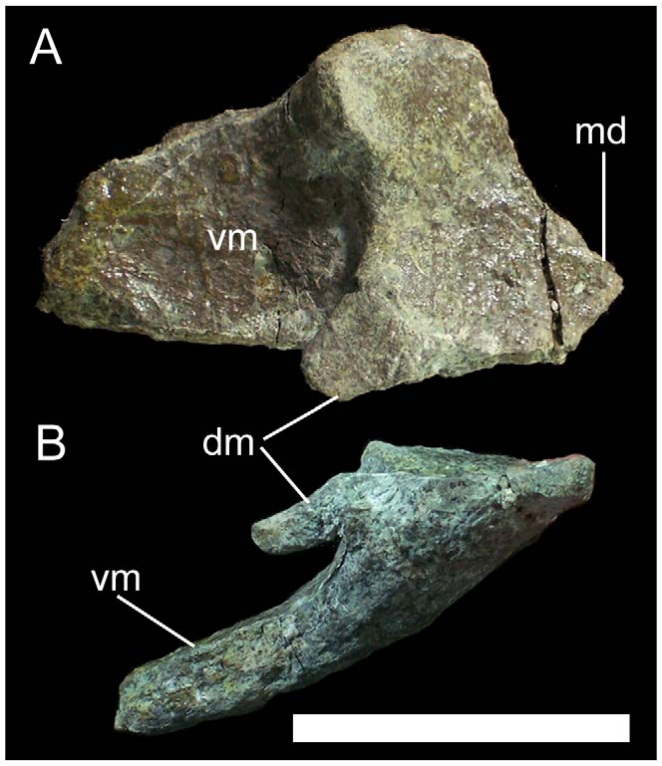
Partial predentary of UMNH VP 20205. Shown in (A) dorsal and (B) right lateral views. *Abbreviations*: *dm*, dorsomedial process; *md*, marginal denticle; *vm*, ventromedial process. Scale bar equals 5 cm.

The left half of the rostral margin with medial processes is the only part of the predentary preserved; the left lateral process is missing, as is the entire right half of the element. There is a single marginal denticle preserved on the oral margin; this is a conical prong projecting rostrodorsally (Fig. 4A). The oral margin is broad and flat and slopes caudoventrally from the marginal denticle to the base of the tab-like dorsomedial process. Ventral to the base of the dorsomedial process, the ventromedial process arises and extends caudoventrally; the dorsal surface of the ventromedial process is gently concave, while the ventral surface is convex (Fig. 4B). Breakage of the ventromedial process renders it impossible to determine whether the process was bifurcated as in other basal iguanodonts such as *Camptosaurus dispar* (YPM PU14553) and *Dakotadon lakotaensis* (SDSM 8656).

**Figure 5:**
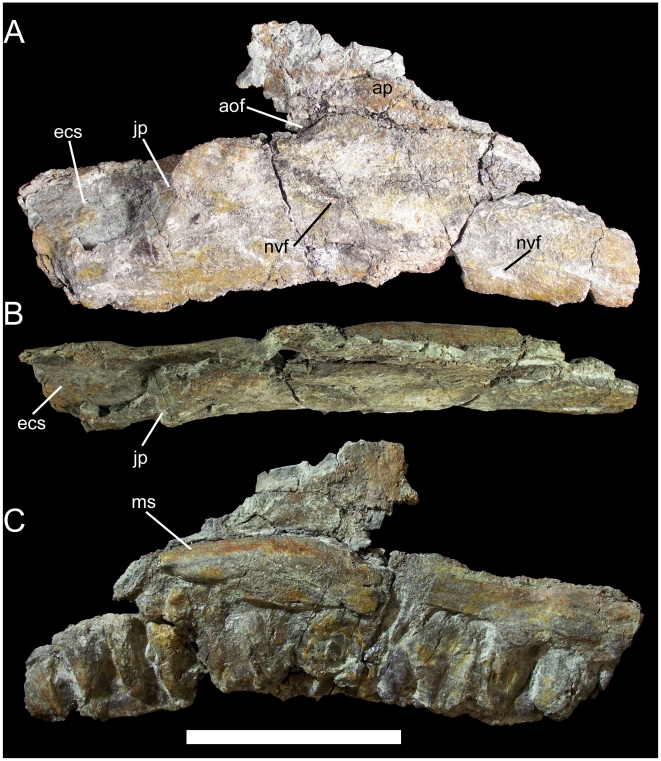
Right maxilla of UMNH VP 20205. Shown in (A) lateral, (B) dorsal, and (C) medial views. *Abbreviations*: *aof*, antorbital fossa; *ap*, ascending process; *ecs*, ectopterygoid shelf; *jp*, jugal process; *ms*, medial shelf; *nvf*, neurovascular foramin. Scale bar equals 10 cm.

The right maxilla is missing its rostral portion and some of the ascending process. The ventral margin of the maxillary tooth row is gently concave in lateral and medial views. The lateral surface of the maxilla is slightly convex and pierced by two prominent neurovascular foramina (Fig. 5A). The jugal process is a sinuous caudolaterally-projecting shelf on the caudolateral margin of the maxilla. The ascending process is incomplete caudally; nevertheless, the shape and relative size of the preserved portion and the length of the broken edge dorsal to the jugal process indicate that the ascending process was rostrocaudally elongate and triangular in lateral view. The rostral margin of the antorbital fossa is a shallow concavity on the ascending process, while the ventral margin of the fossa is a similarly shallow concavity rostrodorsal to the jugal process (Fig. 5A). The positions of these two concave surfaces indicate that the antorbital fossa was probably elliptical in shape and rostrocaudally elongate, as in *Dakotadon lakotaensis* (SDSM 8656), *Theiophytalia kerri* (YPM 1887), and the new taxon *Hippodraco scutodens* (UMNH VP 20208; see below). The ectopterygoid shelf is a broad subrectangular platform caudomedial to the jugal process. The maxilla is somewhat bowed medially in dorsal view (Fig. 5B). There are 14 preserved alveoli. The medial surface of the maxilla immediately dorsal to the alveoli is gently convex, setting it off from the flat medial surface of the ascending process and forming a shelf along the base of the process (Fig. 5C).

**Figure 6:**
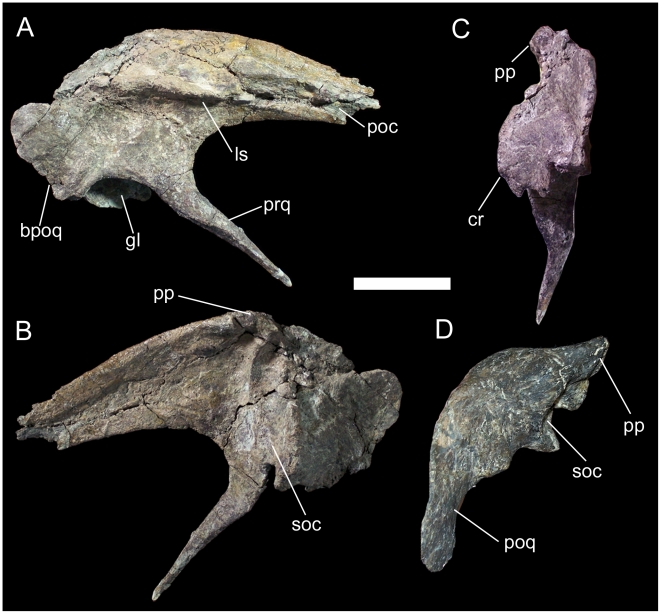
Right squamosal of UMNH VP 20205. Shown in (A) lateral, (B) medial, and (C) caudal views. Left squamosal of *Mantellisaurus atherfieldensis* (NHMUK R5764, holotype) in (D) caudal view. *Abbreviations*: *bpoq*, base of postquadrate process; *cr*, caudal ridge of supraoccipital contact; *gl*, glenoid; *ls*, lateral shelf; *poc*, contact surface of squamosal process of postorbital; *pp*, parietal prong; *prq*, prequadrate process; *soc*, contact surface of supraoccipital. Scale bar equals 5 cm.

The right squamosal is well preserved and quite distinctive. The lateral surface is dominated by a laterally projecting shelf that extends rostrocaudally from the postorbital process to a point directly dorsal to the caudal-most point of the glenoid fossa (Fig. 6A). This shelf probably defines the dorsal boundary of the origin site of *M. adductor mandibulae externus superficialis* [28]. On the postorbital process, this shelf is bounded dorsally by a shallow, rostrocaudally elongate depression, which in turn is bounded dorsally by a fine ridge that terminates at the base of the postorbital process (Fig. 6A). This depression likely represents the lateral contact surface for reception of the squamosal process of the right postorbital. The postorbital process itself tapers rostrally, with a straight ventral margin and convex dorsal margin (Fig. 6A). The process is mediolaterally compressed with a convex lateral surface and concave medial surface, forming a nearly vertical wall dorsal to *M. adductor mandibulae externus superficialis* shelf. This differs from the dorsally broad postorbital processes of the squamosals of other basal iguanodonts, such as *Camptosaurus dispar* (YPM 1880) and *Mantellisaurus atherfieldensis* (NHMUK R5764). A blade-like postorbital process is also present in more basal iguanodontians such as *Zalmoxes robustus* (NHMUK R3402), *Tenontosaurus tilletti* (YPM 5456), and *Dryosaurus altus* (CM 3392). Caudodorsal to *M. adductor mandibulae externus superficialis* shelf, the dorsal margin of the squamosal curves ventrally towards the broken base of the postquadrate process (Fig. 6A). The elongate prequadrate process of the squamosal extends rostroventrally from the rostral margin of the glenoid and tapers to a point at its distal end (Fig. 6A). This process is subtriangular in cross-section, with a convex rostral margin and broad, flat caudal margin. The medial surface of the squamosal dorsal to the glenoid is modified into a caudomedial process as in other basal iguanodonts. The contact surface for the parietal was probably situated on a dorsomedially directed prong, as in *C*. *dispar* and *M*. *atherfieldensis*; this prong is broken off at its base in UMNH VP 20205 (Fig. 6B). Ventral to the base of the parietal prong lies a groove bounded rostrally and caudally by low, well defined ridges (Fig. 6B, C). This groove and the associated ridges form the contact surface for the supraoccipital; in other iguanodontians, this contact surface forms a cup-like depression that is concave in caudal view, as in *C*. *dispar* (YPM 1880) and *M*. *atherfieldensis* (NHMUK R5764; Fig. 6D). In contrast, the supraoccipital contact surface of *Iguanacolossus* is sinuous in caudal view, with a concave dorsal portion and convex ventral portion formed by swelling of the ridge caudal to the aforementioned groove (Fig. 6C). This morphology is here regarded as an autapomorphy of *Iguanacolossus fortis*.

**Figure 7:**
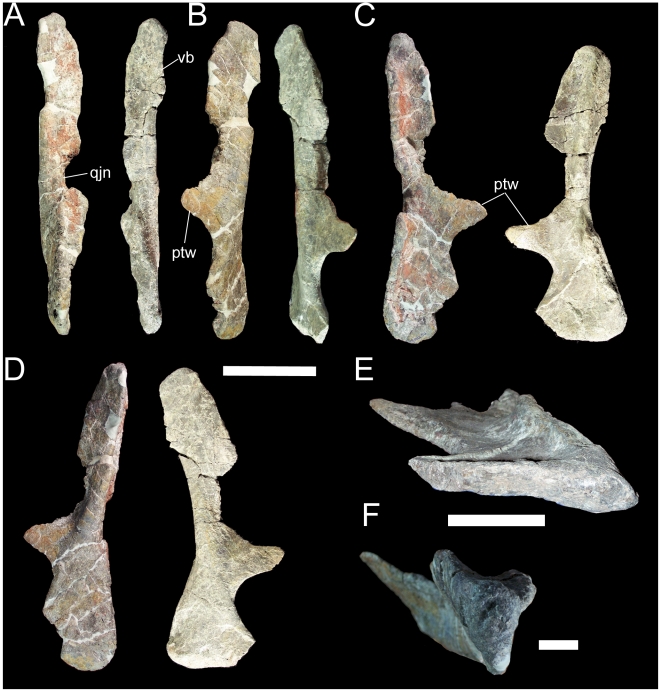
Right and left quadrates of UMNH VP 20205. Shown in (A) lateral, (B) medial, (C) rostral, and (D) caudal views. Left quadrate in (E) ventral view. Right quadrate in (F) dorsal view. *Abbreviations*: *ptw*, pterygoid wing; *qjn*, quadratojugal notch; *vb*, vertical buttress. Scale bar in A–D equals 10 cm; scale bar in E equals 5 cm; scale bar in F equals 1 cm.

The right and left quadrates are nearly complete and only slightly distorted, allowing a full account of the features of the quadrate of *Iguanacolossus*. The quadrate is straight in lateral and medial views (Fig. 7A, B). The caudal margin of the quadrate is flat immediately dorsal to the mandibular condyle and becomes strongly convex towards the dorsal condyle. In rostral and caudal views, the dorsal half of the quadrate is slightly inclined laterally (Fig. 7C, D). The ventral condyle is much broader mediolaterally than it is rostrocaudally (Fig. 7E). The dorsal condyle is roughly subtriangular, with a rounded rostral margin and tapering caudally (Fig. 7F). Ventral to the dorsal condyle, a vertical buttress extends along the caudal margin of the quadrate. The jugal wing of the quadrate bears a broad semi-circular quadratojugal notch (Fig. 7A); the lack of a distinct contact surface for the quadratojugal within the notch itself suggests that a paraquadrate foramen was present. Moreover, the portions of the rostral margin of the jugal wing dorsal and ventral to the notch thicken as they approach the notch, probably signifying the contact surfaces for the quadratojugal. The rostromedially directed pterygoid wing of the quadrate is incomplete along its rostrodorsal margin in both the right and left quadrates of UMNH VP 20205 (Fig. 7B, C, D).

**Figure 8:**
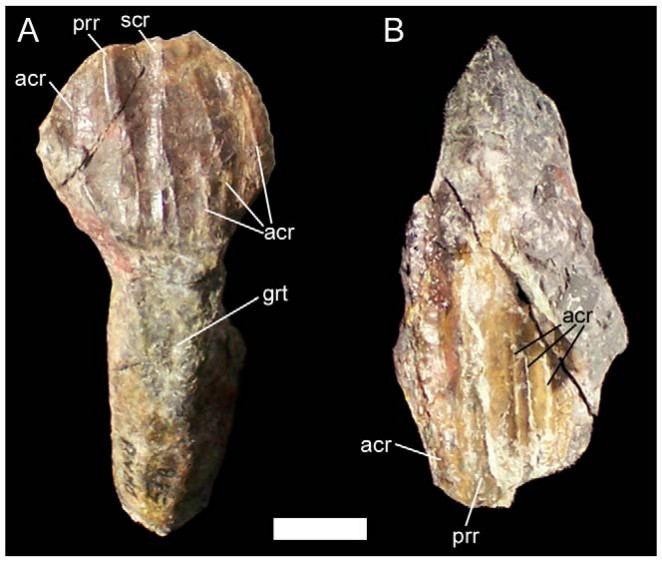
Teeth of UMNH VP 20205. (A) Dentary tooth of UMNH VP 20205 in lingual view. (B) Maxillary tooth of UMNH VP 20205 in labial view. *Abbreviations*: *acr*, accessory ridge; *grt*, groove for replacement tooth; *prr*, primary ridge; *scr*, secondary ridge. Scale bar equals 1 cm.

Two isolated teeth are present in UMNH VP 20205. Both are worn and abraded around the edges, rendering the morphology of the marginal denticles impossible to know. Based upon comparisons with the *in situ* dentary and maxillary dentitions of *Camptosaurus dispar* (YPM 1886) and *Dakotadon lakotaensis* (SDSM 8656), the tooth with the broader, shield-shaped crown is regarded as a dentary tooth, while the more lozenge-shaped crown is treated as a maxillary tooth. The lingual surface of the dentary tooth crown is traversed by no fewer than six ridges of varying prominence, the most prominent of which is probably the primary ridge (Fig. 8A). On one side of the primary ridge is a very faint accessory ridge that probably arose from a marginal denticle. On the other side of the primary ridge is a ridge of similar prominence and that could be the secondary ridge. There are three fainter accessory ridges between the possible secondary ridge and the crown margin. The lingual surface of the tooth root bears a shallow groove in which the next replacement tooth in the series would have rested. The labial surface of the maxillary tooth crown exhibits five ridges, one of which, the primary ridge, is much more prominent than the others (Fig. 8B). On one side of the primary ridge there is a single faint accessory ridge, while on the other side of the primary ridge there are three accessory ridges.

**Figure 9:**
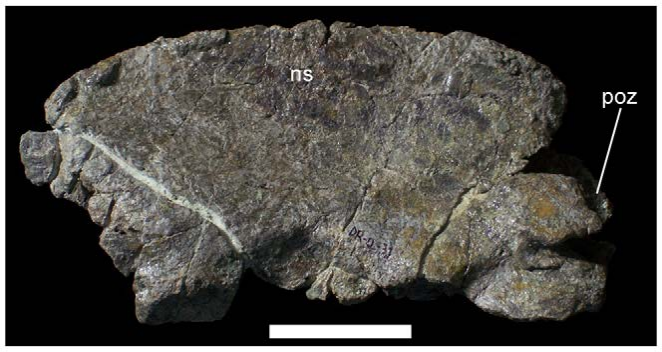
Axial neural arch of UMNH VP 20205. Shown in left lateral view. *Abbreviations*: *ns*, neural spine; *poz*, postzygapophysis. Scale bar equals 5 cm.

The cervical vertebrae are represented by only the neural arch of the axis. The axial neural spine is mediolaterally compressed and dorsally convex in lateral view (Fig. 9), forming a large blade-like structure similar to those of styracosternans such as *Iguanodon bernissartensis* (IRSNB 1639) and *Corythosaurus casuarius* (CM 9461), and quite dissimilar from the sloping, dorsally concave axial neural spines of more basal iguanodontians such as *Tenontosaurus tilletti* (YPM 5456), *Dryosaurus altus* (CM 3392), and *Camptosaurus dispar* (USNM 5473; YPM 1877).

**Figure 10:**
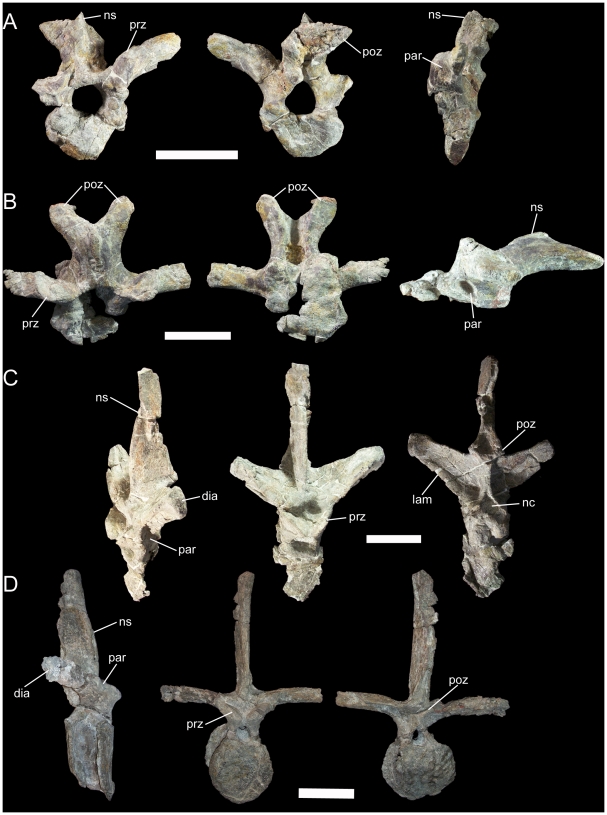
Representative dorsal vertebrae of UMNH VP 20205. (A) Dorsal 1 in cranial, caudal, and left lateral views. (B) Dorsal 2 in dorsal, ventral, and left lateral views. (C) Middle dorsal in left lateral, cranial, and caudal views. (D) Caudal dorsal in right lateral, cranial, and caudal views. *Abbreviations*: *dia*, diapophysis; *lam*, lamina; *nc*, neural canal; *ns*, neural spine; *par*, parapophysis; *poz*, postzygapophysis; *prz*, prezygapophysis. Scale bars equal 10 cm.

**Figure 11:**
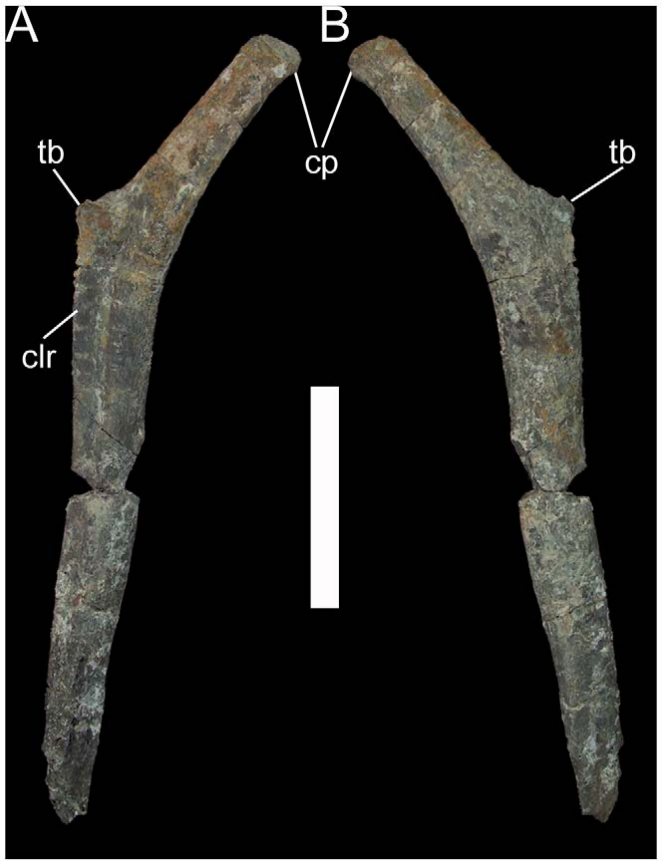
Right dorsal rib of UMNH VP 20205. Shown in (A) cranial and (B) caudal views. *Abbreviations*: *cp*, capitulum; *clr*, craniolateral ridge; *tb*, tuberculum. Scale bar equals 10 cm.

Thirteen partial to nearly complete dorsal vertebrae are preserved in UMNH VP 20205. Many of these dorsals are incomplete or have suffered severe distortion; thus, their positions in the skeletal reconstruction of UMNH VP 20205 (Fig. 3A) should be regarded as conjectural. Comparison of UMNH VP 20205 to basal iguanodonts for which complete and articulated dorsal series are known, such as “*Camptosaurus*” *aphanoecetes* (CM 11337) [29] and *Iguanodon bernissartensis* (IRSNB 1534) [30], provide a guide to the probable order of the dorsal vertebrae. The most complete and undistorted representative dorsals are described here and together provide a fairly complete picture of the dorsal series of *Iguanacolossus*; however, even in these otherwise well preserved dorsals, the centra are extremely compressed craniocaudally or badly damaged. Two of the dorsals are identifiable as cranial dorsals, probably dorsals 1 and 2, as indicated by their short, spur-like neural spines; prezygapophyses situated on the transverse processes; elongate, arching postzygapophyses; and parapophyses situated near the base of the transverse processes on the neural arches rather than on the centra as in cervical vertebrae (Fig. 10A, B). The next selected dorsal is closer to the middle of the series, probably belonging between dorsals 4 and 10. The neural spine of this vertebra is much taller than those of dorsals 1 and 2 and tapers towards its apex, with strongly convex cranial and caudal margins (Fig. 10C). The dorsolaterally-directed transverse processes are dorsoventrally deep at their bases, with a thick lamina extending from the dorsal margin of the caudal face of the centrum to the diapophyses. The parapophyses are cup-like depressions at the bases of the transverse processes; the diapophyses are rugose surfaces at the ends of the transverse processes. The prezygapophyses are mediodorsally-directed tabs cranial to the parapophyses, while the postzygapophyses arise from the caudal margin of the neural spine and face ventrolaterally (Fig. 10C). A more caudal dorsal is likely from between dorsals 9 and 13, as indicated by the reduced parapophyses relative to the middle dorsal described above; in *Iguanodon bernissartensis*, the parapophyses become progressively smaller towards the caudal end of the dorsal series [30]. This caudal dorsal is otherwise quite similar to the middle dorsal, with a dorsally tapering neural spine (Fig. 10D). The dorsal ribs are typical of basal iguanodonts, with an elongate, prong-like capitulum and small, rugose tuberculum (Fig. 11A, B). The cranial surface of the dorsal rib is slightly concave, with a ridge along the craniolateral margin; the caudal surface is slightly convex.

**Figure 12:**
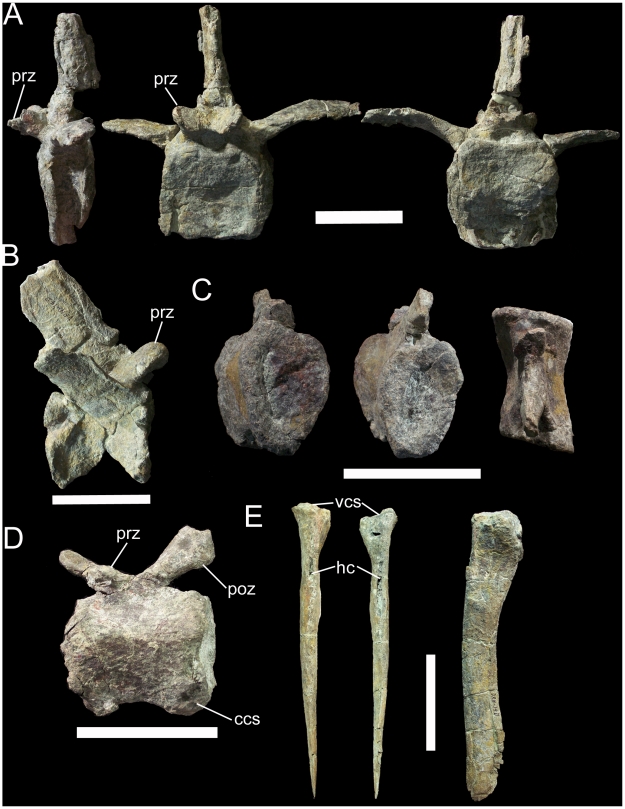
Representative caudal vertebrae and chevron of UMNH VP 20205. (A) Cranial caudal in left lateral, cranial, and caudal views. (B) Cranial to middle caudal in right lateral view. (C) Middle to distal caudal in cranial, caudal, and dorsal views. (D) Middle to distal caudal in left lateral view. (E) Cranial chevron in cranial, caudal, and left lateral views. *Abbreviations*: *ccs*, contact surface for associated chevron; *hc*, haemal canal; *poz*, postzygapophysis; *prz*, prezygapophysis; *vcs*, contact suface for associated vertebra. Scale bars equal 10 cm.

Five caudal vertebrae and five chevrons are preserved in UMNH VP 20205. The single cranial caudal is distinguished from the dorsals by its lack of parapophyses (Fig. 12A). The transverse processes curve ventrolaterally from the base of the neural arch (Fig. 12A). The prezygapophyses are expanded into finger-like processes dissimilar to the simple tab-like prezygapophyses of the middle and caudal dorsals described above, while the postzygapophyses are missing. The next caudal is somewhat more distal, with a neural spine strongly inclined caudally (Fig. 12B). The prezygapophyses are similar to those of the more cranial caudal; the postzygapophyses face ventrolaterally. The remaining three caudals are very similar to each other and come from the middle to distal portion of the caudal series. The cranial and caudal faces of the centra are wedge-shaped, being broader across their dorsal margins and narrowing towards their ventral margins (Fig. 12C). The centra are hourglass-shaped in dorsal view (Fig. 12C). The most complete middle-distal caudal shows that the prezygapophyses face dorsomedially and are placed on elongate prongs that project craniodorsally (Fig. 12D). The postzygapophyses face ventrolaterally and are round, flat surfaces on the caudoventral margin of the neural spine. Two facets on the caudoventral margin of the centrum indicate the contact surface for the chevron associated with this vertebra. The most complete chevron exhibits a laterally-expanded proximal end that would have contacted the centrum of the associated vertebra (Fig. 12E). The haemal canal is an elliptical opening immediately distal to the proximal contact surface. The shaft of the chevron curves caudally along its length.

**Figure 13:**
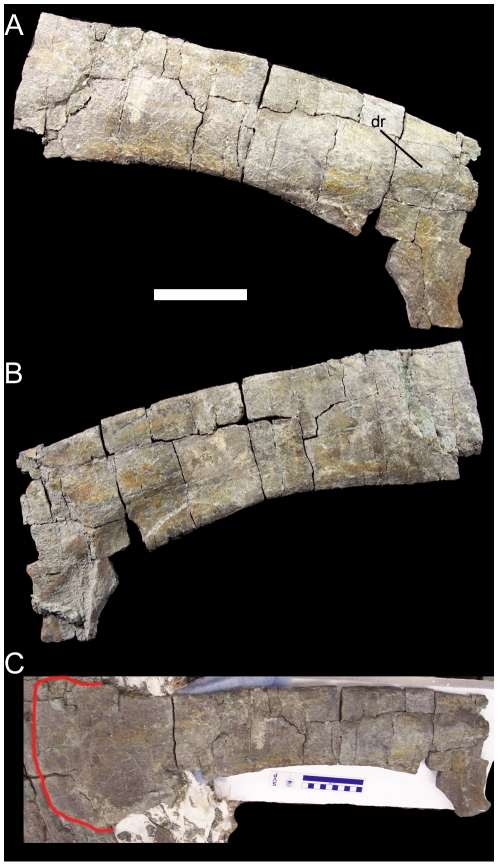
Right scapula of UMNH VP 20205. Shown in (A) lateral, (B) medial, and (C) lateral view with scapular blade held against the detached caudal end of the scapula (caudal end outlined in red). *Abbreviations*: *dr*, deltoid ridge. Scale bars equal 10 cm.

The right scapula of UMNH VP 20205 is nearly complete, though it is lacking most of its cranial end. The scapular blade is gently convex along its dorsal margin and concave along its ventral margin (Fig. 13A, B). The caudal end is preserved but is closely appressed to several dorsal ribs, making preparation difficult; when the caudal end is held against the rest of the scapula, it becomes clear that the dorsal and ventral margins diverged caudally to form a broad paddle-like expansion of the caudal end of the scapula (Fig. 13C). The deltoid ridge is a low eminence that extends along the lateral surface of the scapula before disappearing just caudal to the base of the scapular blade (Fig. 13A).

**Figure 14:**
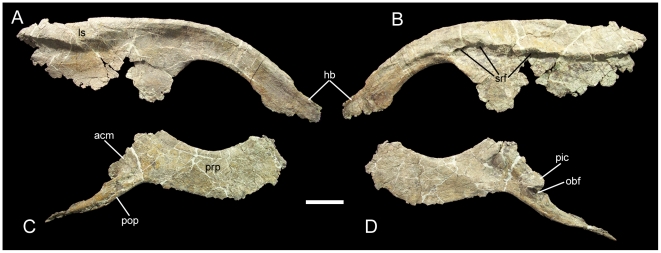
Pelvic elements of UMNH VP 20205. Right ilium of UMNH VP 20205 in (A) lateral and (B) medial views. Right pubis in (C) lateral and (D) medial views. *Abbreviations*: *acm*, acetabular margin; *hb*, horizontal boot of preacetabular process; *ls*, lateral swelling; *obf*, obturator foramen; *pic*, contact surface for pubic peduncle of ischium; *pop*, postpubic process; *prp*, prepubic process; *srf*, sacral rib facet. Scale bar equals 10 cm.

The pelvis of UMNH VP 20205 is represented by the well preserved right ilium and pubis. The right ilium is incomplete in the acetabular region and at the caudal end of the postacetabular process. The preacetabular process is strongly curved ventrally and terminates in a cranially expanded horizontal boot (Fig. 14A, B). The dorsal margin of the ilium is straight. Dorsal to the ischial peduncle, the lateral surface of the ilium bulges outwards to form a lateral swelling (Fig. 14A). This lateral swelling is offset from the lateral surface of the ilium immediately below it. The dorsal margin of the ilium dorsal to the ischial peduncle is not thickened or modified in any fashion. The lateral swelling of *Iguanacolossus* prompted a reconsideration of a similar structure in *Cedrorestes* (see below). On its medial surface, the ilium bears a shelf with a series of rounded facets along its ventral margin (Fig. 14B); these facets form the contact surface for the right sacral ribs.

The right pubis is missing the iliac peduncle. The ventral margin of the prepubic process is somewhat fragmented, but the bone is extremely thin along this margin, so it is likely that little of the prepubic process is missing. The prepubic process has parallel dorsal and ventral margins, lacking expansion of its cranial end; the dorsal margin is strongly concave while the ventral is strongly convex (Fig. 14C). This is similar to the prepubic processes of more basal iguanodonts such as *Camptosaurus dispar* (YPM 7334) and “*Camptosaurus*” *aphanoecetes* (CM 11337), and different from the cranially expanded prepubic processes of more derived iguanodonts such as *Iguanodon bernissartensis* (IRSNB 1534) and *Ouranosaurus nigeriensis* (MNHN GDF 300). The postpubic process is relatively short compared to the prepubic and tapers to a blunt point (Fig. 14C). This differs from the elongate and distally expanded postpubic processes of more basal iguanodonts such as *Dryosaurus altus* (CM 3392) and *Camptosaurus dispar* (YPM 1878), but is similar to the postpubic processes of more derived iguanodonts. This combination of plesiomorphic and derived features in the pubis is unique to UMNH VP 20205 and is diagnostic for *Iguanacolossus fortis*. A curved flange originates on the caudal margin of the pubis at the base of the postpubic process; this flange, together with a small eminence on the caudal margin of the postpubic process, partially encloses the obturator foramen and forms the contact surface for the pubic peduncle of the right ischium (Fig. 14D). Dorsal to the flange is the smooth, gently curved acetabular margin of the pubis.

**Figure 15:**
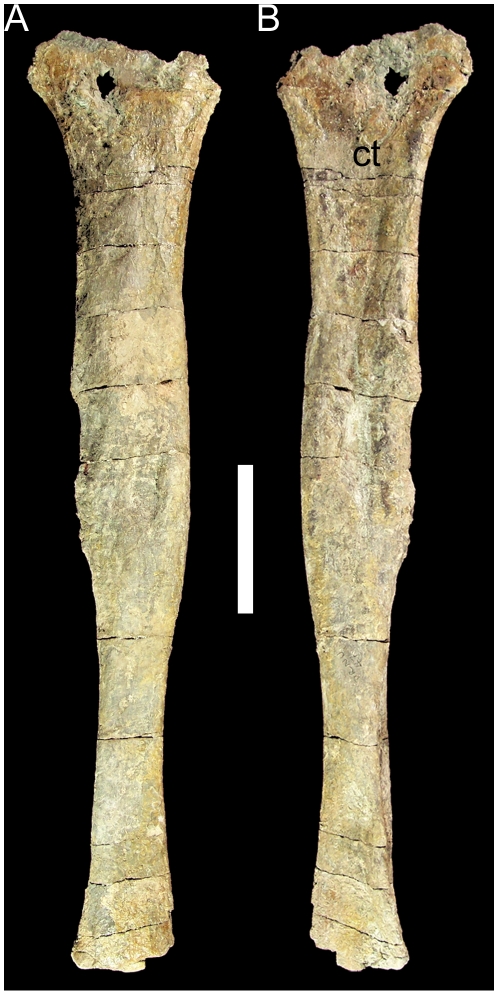
Left fibula of UMNH VP 20205. Shown in (A) lateral and (B) medial views. *Abbreviations*: *ct*, contact surface for fibular process of tibia. Scale bar equals 10 cm.

The only elements recovered from the hindlimb of UMNH VP 20205 are the left fibula and two metatarsals. The fibula is missing its distal end but is otherwise intact. The proximal end and preserved portion of the distal end are both craniocaudally expanded (Fig. 15A, B). The medial side of the proximal end is concave to receive the fibular process of the left tibia (Fig. 15B). The shaft of the fibula is of almost uniform thickness immediately distal to the proximal end until approximately halfway down the shaft, at which point the shaft becomes considerably narrower.

**Figure 16:**
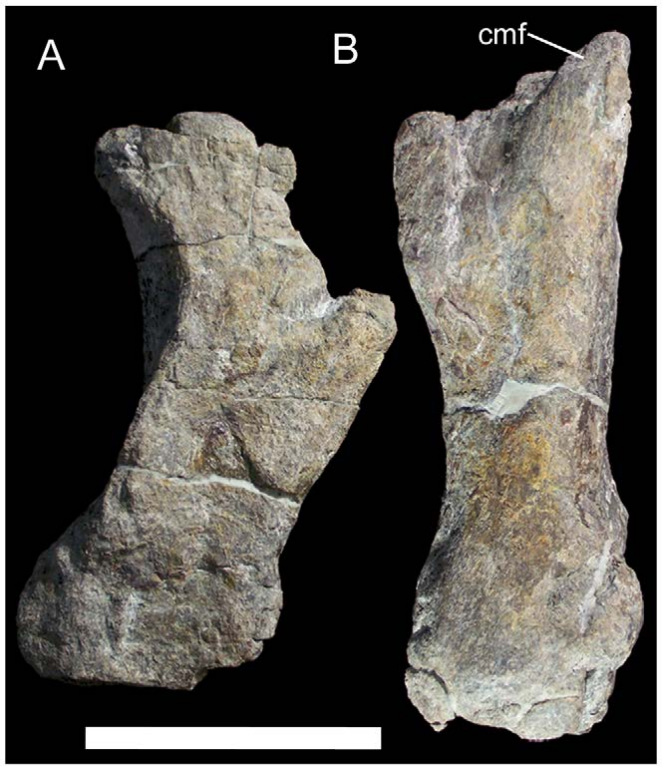
Metatarsals of UMNH VP 20205. Right metatarsals IV (A) and III (B) of UMNH VP 20205 in dorsal view. *Abbreviations*: *cmf*, craniomedial flange. Scale bar equals 10 cm.

Based upon comparisons with articulated metatarsi of *Camptosaurus dispar* (YPM 1877) and *Iguanodon bernissartensis* (IRSNB 1534), the two metatarsals are identified as the right MT III and IV. Both metatarsals have suffered breakage and abrasion of their articular surfaces and some degree of crushing. MT IV is strongly curved laterally in dorsal view (Fig. 16A). MT III is straight in dorsal view with a craniomedial flange arising from its proximal end that would have overlapped the proximal end of the right MT II (Fig. 16B).

### Reassessment of *Cedrorestes crichtoni*

*Cedrorestes crichtoni* was named by Gilpin et al. [14], and shortly thereafter was considered a probable subjective junior synonym of *Planicoxa venenica* by Kirkland and Madsen [20]. A recent reassessment of *P*. *venenica* revealed that the holotype ilium (DMNH 42504) differs from that of *Cedrorestes* in having a convex dorsal margin and both taxa are reservedly treated as viable [ATM, in review].

*Cedrorestes crichtoni* was originally distinguished from other basal iguanodonts by the combination of two features of the ilium, after Gilpin et al. [14: page 82]: ‘deep, iguanodontid-like ilium, but having a hadrosaurid-like prominent lateral process (“antitrochanter”) dorsal to the ischial peduncle’. The first feature is difficult to assess. The composition and even the existence of a monophyletic Iguanodontidae are highly equivocal [31]–[34, this paper] and thus it is not clear to which taxa *Cedrorestes* is being compared; furthermore, the statement itself is a subjective qualitative version of what could be expressed as a continuous quantitative character, i.e., the ratio between the craniocaudal length of the postacetabular process and the greatest depth of the process.

**Figure 17:**
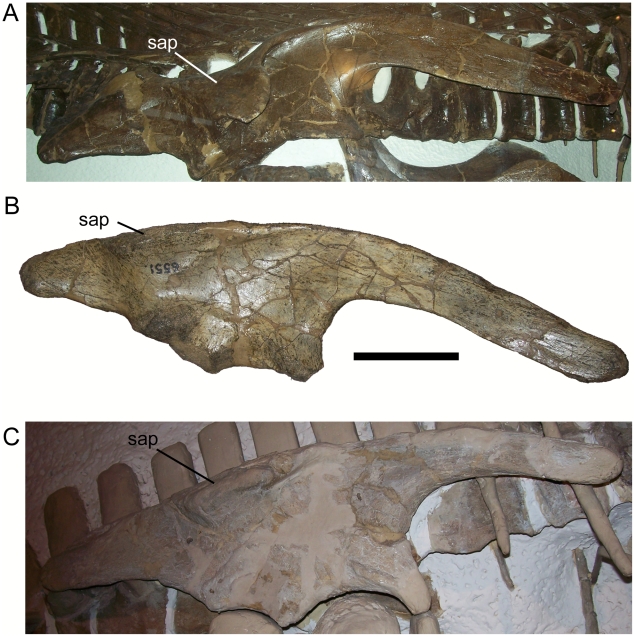
Supraacetabular processes of other iguanodonts. Right ilia of (A) *Corythosaurus casuarius* (AMNH 5338, paratype); (B) *Gilmoreosaurus mongoliensis* (AMNH 6551, holotype); and (C) *Claosaurus agilis* (YPM 1190, holotype, image copyright YPM) in lateral view. *Abbreviations*: *sap*, supraacetabular process. Scale bar in B equals 10 cm.

**Figure 18:**
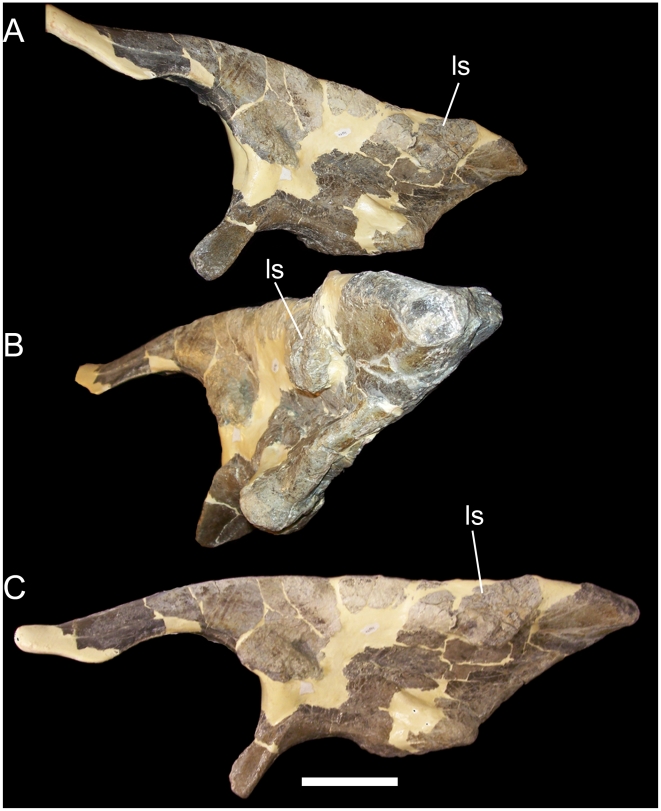
Left ilium of *Cedrorestes crichtoni* (DMNH 47994, holotype). Shown in (A) craniolateral, (B) caudomedial, and (C) lateral views. *Abbreviations*: *ls*, lateral swelling. Scale bar in C equals 10 cm.

The second feature, the “hadrosaurid-like prominent lateral process” or supraacetabular process, is also problematic and another plausible interpretation can be made. The supraacetabular process of DMNH 47994 differs from those of hadrosaurids in being restricted to the lateral surface of the ilium. In hadrosaurids such as *Corythosaurus casuarius* (AMNH 5338; USNM 15493), the pendant supraacetabular process is formed from a lateral eversion of the dorsal margin of the ilium, such that the lateral surface of the supraacetabular process is continuous with the dorsal margin of the ilium (Fig. 17A). This also the case in some close outgroups of Hadrosauridae [35], such as *Gilmoreosaurus mongoliensis* (AMNH 6551; Fig. 17B) and *Claosaurus agilis* (YPM 1190; Fig. 17C), in which the supraacetabular process is present but is not pendant. In contrast, the supposed supraacetabular process of DMNH 47994 is restricted to the lateral surface of the ilium; the dorsal margin of the ilium displays no sign of the eversion noted in *Gilmoreosaurus*, *Claosaurus*, and *Corythosaurus* (Fig. 18A). Furthermore, the damaged ventral margin of the supraacetabular process of DMNH 47994 is composed of filler and the lateral surface of the ilium ventral to the supraacetabular process is crushed, forming a depression (Fig. 18B); this filler and crushing of the lateral surface of the ilium might make the supraacetabular process seem more prominent than it would have normally appeared. The “supraacetabular process” of *Cedrorestes* can thus be interpreted as a swelling restricted to the lateral surface of the ilium, similar to the structure described above in *Iguanacolossus*; this structure is best characterized as a lateral swelling. The ilia of *Cedrorestes* and *Iguanacolossus* differ in the curvature of the preacetabular process, it being much more pronounced in the latter, and in the shape of the lateral swelling; in *Cedrorestes*, the angle formed by the cranial and caudal margins of the swelling is much more acute than in *Iguanacolossus* (Fig. 18C). However, without additional specimens of these two taxa, it cannot be determined whether these differences are the result of distortion, ontogeny, or individual variation.

Considering the new interpretation presented above, it could be argued that UMNH VP 20205 is better referred to *Cedrorestes crichtoni* than made the holotype of a new taxon. However, there are several reasons for keeping DMNH 47994 and UMNH VP 20205 in separate taxa. There is some stratigraphic disparity between the two specimens, with DMNH 47994 coming from the Poison Strip Member [20] (originally reported as coming from the upper Yellow Cat [14]) and UMNH VP 20205 coming from the lower part of the Yellow Cat Member. Stratigraphy aside, three conditions support the creation of a new taxon for UMNH VP 20205. First, the ilium is the only element shared between the holotype of *Cedrorestes* and the much more complete holotype of *Iguanacolossus*, precluding a more detailed comparison between the two taxa. Second, as described above and shown in Figure 18, the ilium of DMNH 47994 is damaged in several places, including the region of the lateral swelling or supraacetabular process. It is therefore difficult to diagnose *Cedrorestes crichtoni* and to maintain confidence in any interpretation of its anatomy, and further discoveries might vindicate the original interpretation of Gilpin et al. [14]; it might be that the supraacetabular process is simply crushed and closely appressed to the lateral surface of the ilium. *Cedrorestes* is coded as having a lateral swelling for the purposes of the current phylogenetic analysis. Third, in no iteration of the phylogenetic analysis, including the reduced consensus tree, do *Cedrorestes* (coded based upon DMNH 47994) and *Iguanacolossus* (coded based upon UMNH VP 20205) exhibit a sister-taxon relationship. Given these lingering uncertainties over the nature of DMNH 47994, the most prudent course is to create a new taxon to receive the more complete UMNH VP 20205, which can be readily distinguished from other basal iguanodonts based upon an autapomorphy and unique combination of characters.

### 2. Upper Yellow Cat Iguanodont

#### Systematic Paleontology (as for *Iguanacolossus fortis*)

*Hippodraco scutodens* gen. et sp. nov.

#### ZooBank LSID for genus

urn:lsid:zoobank.org:act:6E4ADED6-BEE0-4EE7-94D9-F3506DC333D1.

#### ZooBank LSID for species

urn:lsid:zoobank.org:act:D42E32C2-CFAE-479C-BCA1-C9EB576BC603.

#### Holotype

UMNH VP 20208, the associated skeleton of a single individual, including a nearly complete skull and partial postcranium.

#### Specific Diagnosis (as for genus by monotypy)

Basal styracosternan diagnosed by a single autapomorphy: dentary tooth row strongly offset medially by a rounded lateral shelf that extends along the dorsolateral margin of the dentary from the first alveolus to the base of the coronoid process and slopes ventromedially to contact the labial margin of the tooth row. Also distinguished from all other iguanodontians except *Theiophytalia kerri* by the following combination of characters: finely striated flange that extends from the caudoventral margin of the jugal, projecting caudal to the jugal-quadratojugal contact; and lack of a gap (paraquadrate foramen) between quadratojugal and quadrate.

#### Locality and Horizon

Andrew's Site (discovered by ARCM in 2004), UMNH VP locality 1207, northeast of Arches National Park, Grand County, Utah; exact locality information is on file at the Utah Geological Survey and Utah Museum of Natural History. Collected in the upper portion of the Yellow Cat Member of the Cedar Mountain Formation (upper Barremian-lowermost Aptian, Lower Cretaceous) [16], [17], [20], [21].

#### Etymology

*Hippodraco*, from the transliterated Greek *hippos* (horse) and the Latin *draco* (dragon), in reference to the long and low overall shape of the skull, grossly resembling that of a horse; *scutodens*, from the Latin *scutum* (oblong shield) and *dens* (tooth), in reference to the shape of the dentary tooth crowns. The gender of the genus is masculine. The intended meaning of the binomen is ‘shield-toothed horse-dragon’.

### Description

**Figure 19:**
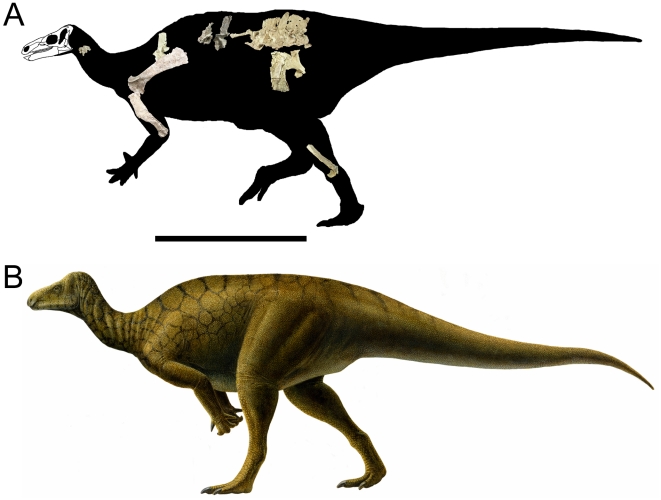
Reconstruction and restoration of *Hippodraco scutodens*. (A) Skeletal reconstruction of *Hippodraco scutodens*, showing the known elements of UMNH VP 20208 (the right scapula, right humerus, right femur, and right tibia have been reversed for the purposes of reconstruction). (B) Life restoration of *Hippodraco scutodens* by Lukas Panzarin. Scale bar in A equals 1 meter.

**Figure 20:**
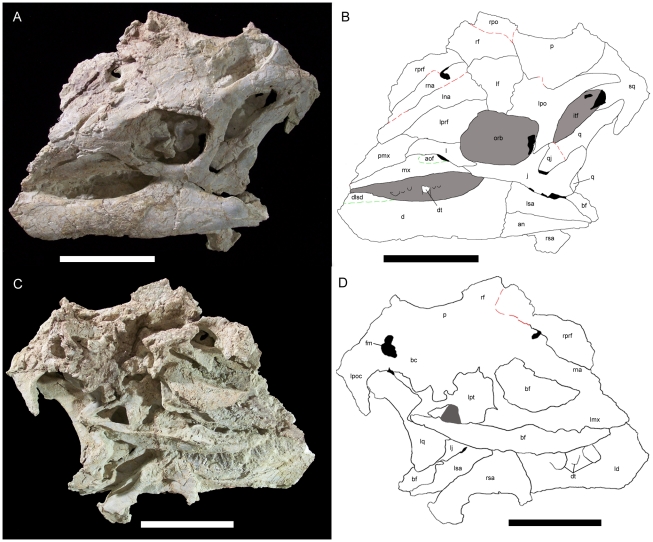
Skull of *Hippodraco scutodens*. (A) Skull of UMNH VP 20208, holotype of *Hippodraco scutodens*, in left lateral view. (B) Tracing of the skull of UMNH VP 20208 in left lateral view. Bones are white, matrix is grey, and empty spaces are black. Sutures and bone outlines are represented by solid black lines, ambiguous sutures by red dashed lines, and surficial features of the bones by green dashed lines. (C) Skull of UMNH VP 20208 in left medial view. (D) Tracing of the skull of UMNH VP 20208 in left medial view. Bones are white, matrix is grey, and empty spaces are black. Sutures and bone outlines are represented by solid black lines, ambiguous sutures by red dashed lines. *Abbreviations*: *an*, angular; *aof*, antorbital fossa; *bc*, braincase; *bf*, bone fragment; *d*, dentary; *dlsd*, dorsolateral shelf of dentary; *dt*, dentary tooth; *fm*, foramen magnum; *itf*, infratemporal fenestra; *j*, jugal; *l*, lacrimal; *ld*, left dentary; *lf*, left frontal; *lj*, left jugal; *lmx*, left maxilla; *lna*, left nasal; *lpo*, left postorbital; *lpoc*, left paroccipital process; *lprf*, left prefrontal; *lpt*, left pterygoid; *lq*, left quadrate; *lsa*, left surangular; *mx*, maxilla; *orb*, orbit; *p*, parietal; *pmx*, premaxilla; *q*, quadrate; *qj*, quadratojugal; *rf*, right frontal; *rna*, right nasal; *rpo*, right postorbital; *rprf*, right prefrontal; *rsa*, right surangular; *sq*, squamosal. Scale bars equal 10 cm.

**Figure 21:**
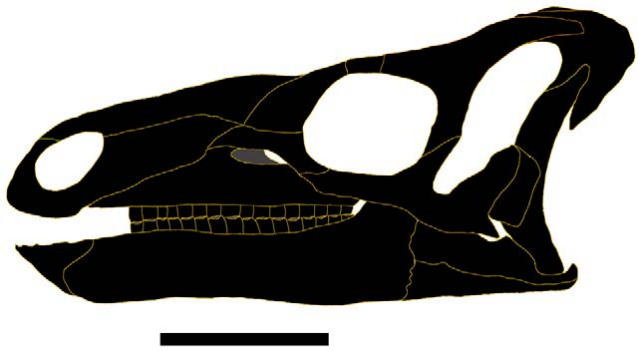
Reconstruction of the skull of UMNH VP 20208. Shown in left lateral view. Scale bar equals 10 cm.

Measurements of UMNH VP 20208 are given in Table 2. *Hippodraco* is restored as a rather small and gracile animal (∼4.5 meters in length) (Fig. 19), although it is necessary to note that the ontogenetic stage of the holotype and only known specimen is ambiguous. The proportionately large orbit suggests that it is immature. The skull of *Hippodraco* is nearly complete on its left side, though it has suffered mediolateral distortion such that the dorsal surface of the skull roof is visible in left lateral view, and many elements of the right side are missing or badly fragmented and obscured (Fig. 20A, C). However, most of the elements of the left side are present and numerous sutures are visible on the left lateral surface (Fig. 20B), facilitating detailed description of much of the cranial anatomy. In contrast, the medial surface of the skull is comparatively poorly preserved (Fig. 20C, D); moreover, large bone fragments that might represent parts of the right maxilla and dentary obscure the medial aspects of many bones of the left side. Note that the skull reconstruction presented in Figure 21 is intended merely as an idealized depiction of how the skull might have appeared during life; it is not meant to be used for detailed comparison with other basal iguanodonts or to code *Hippodraco* in any future phylogenetic analyses.

**Table 2:** Measurements of UMNH VP 20208, the holotype of *Hippodraco scutodens*.

Element	Measurement
Right surangular, total rostrocaudal length	9.4
Left maxilla, greatest preserved rostrocaudal length	13.2
Left maxilla, greatest dorsoventral height	3.4
Left maxilla, greatest rostrocaudal length of antorbital fossa	2.7
Parietal, total rostrocaudal length	7.0
Parietal, rostrocaudal length of sagittal crest	2.0
Parietal, minimum width	3.5
Left postorbital, total rostrocaudal length along dorsal margin	12.0
Left postorbital, rostrocaudal length of squamosal process	6.9
Left jugal, total rostrocaudal length along ventral margin	11.8
Left quadrate, greatest mediolateral width of ventral condyle	∼2.5
Left quadrate, greatest preserved dorsoventral height	11.4
Left orbit, greatest rostrocaudal width	7.6
Left orbit, greatest dorsoventral height	6.0
Left infratemporal fenestra, greatest dorsoventral height	9.8
Left supratemporal fenestra, greatest rostrocaudal length	6.4
Left supratemporal fenestra, greatest mediolateral width	3.3
Left sternal, total craniocaudal length	16.5
Left sternal, greatest mediolateral width	5.9
Right scapula, total craniocaudal length	45.5
Right scapula, greatest dorsoventral depth of cranial portion, from apex of acromion process to apex of scapular labrum	13.2
Right scapula, greatest dorsoventral depth of caudal end	13.1
Right humerus, total proximodistal length	32.3
Right humerus, proximodistal length of the deltopectoral crest	13.0
Left MT II, total proximodistal length	∼16
Left MT III, total proximodistal length	∼21
Left MT IV, total proximodistal length	∼14
Left DII, p1, total proximodistal length	5.2
Left DII, p2, total proximodistal length	3.4
Left DII, p3, total proximodistal length	8.2
Left DIII, p1, total proximodistal length	6.5
Left DIII, p3, total proximodistal length	1.8
Left DIII, p4, total proximodistal length	8.3
Left DIV, p1, total proximodistal length	5.8
Left DIV, p5, total proximodistal length	6.6

Measurements are given in centimeters.

**Figure 22:**
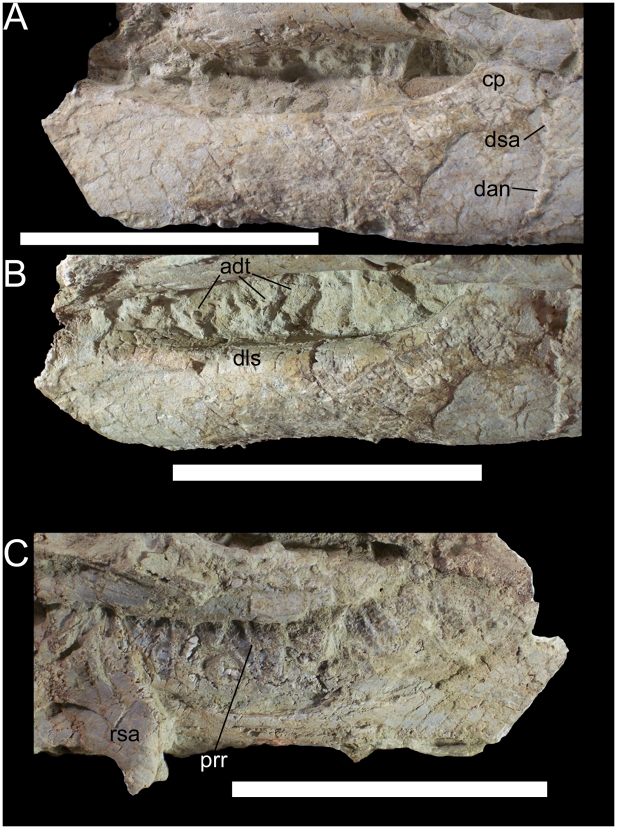
Left dentary and dentary teeth of UMNH VP 20208. Shown in (A) lateral, (B) dorsolateral, and (C) medial views. *Abbreviations*: *adt*, active dentary teeth; *cp*, coronoid process; *dan*, suture between dentary and angular; *dls*, dorsolateral shelf; *dsa*, suture between dentary and surangular; *prr*, primary ridge; *rsa*, right surangular. Scale bars equal 10 cm.

The predentary and probably much of the right dentary are missing; the left dentary is missing its rostral-most portion but is otherwise well preserved. The rostral and ventral margins of the dentary are parallel; as far as can be ascertained, the dentary does not appear to taper or deepen rostrally. Although the rostral ramus is incomplete, the curvature of the ventral margin implies that the rostral ramus was ventrally inflected, though to what degree relative to other basal iguanodonts is impossible to tell. The dentary tooth row is straight along its dorsal margin in lateral view (Fig. 22A). In dorsolateral view, the tooth row is straight for much of its length but curves laterally near its caudal end and merges with the base of the coronoid process (Fig. 22B). The coronoid process of UMNH VP 20208 is overlain by the left jugal, and thus its morphology and orientation are nebulous. The dentary tooth row has a very pronounced medial offset relative to the lateral surface of the dentary due to a broad shelf lateral to the tooth row that extends from the rostral-most preserved portion of the dentary to the base of the coronoid process (Fig. 22B). This shelf is strongly convex along its dorsolateral margin, becoming gently concave as it slopes medially towards the tooth row. This shelf is unique to UMNH VP 20208 and is an autapomorphy of *Hippodraco scutodens*. In other basal iguanodonts, such as *Mantellisaurus atherfieldensis* (NHMUK R5764) and the otherwise similar *Theiophytalia kerri* (YPM 1887), the lateral surface of the dentary is gently convex from the ventral to the dorsal margin; there is medial offset of the tooth row, but it is not as pronounced as in *Hippodraco* nor is there a broad shelf lateral to the tooth row. The dentary of UMNH VP 20208 does not appear to be pathological, nor is it likely that the dentary shelf is a product of plastic deformation; that the dorsal surface of the skull roof is visible in lateral view indicates that the skull was flattened by compression operating in a mediolateral direction. If the dentary had been crushed in the same fashion, it would be expected to have flattened out like the rest of the skull rather than to have curled over on itself to form a lateral shelf. Therefore, the lateral shelf is interpreted as an actual morphological feature of *Hippodraco*.

The dentary teeth of UMNH VP 20208 are *in situ* though not well preserved, having suffered cracking and fragmentation like much of the bone surface of the skull. The morphology of the marginal denticles cannot be determined. There is a single replacement tooth per tooth position (Fig. 22C). Although the labial surfaces of the tooth crowns are largely obscured by matrix, it is apparent that only one tooth in each position participated in the occlusal plane (Fig. 22B). Unworn crowns are oblong, broad, and shield-shaped in lingual view (Fig. 22C). Due to damage to the unworn crowns, the number and morphologies of any secondary and accessory ridges cannot be ascertained; however, a distally offset primary ridge is visible on several crowns (Fig. 22C).

**Figure 23:**
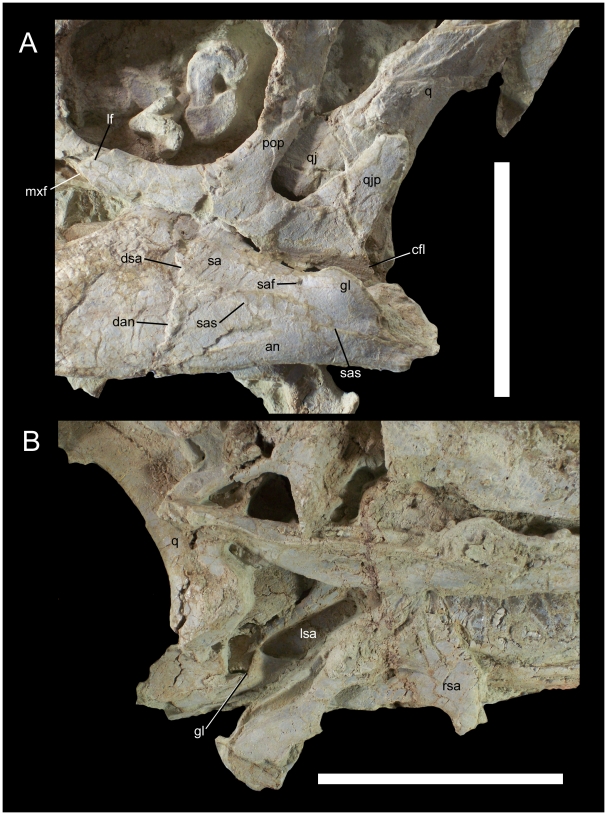
Caudal region of the mandible and caudoventral region of the skull of UMNH VP 20208. Shown in (A) left lateral and (B) left medial views. *Abbreviations*: *an*, angular; *cfl*, caudal flange; *dan*, suture between dentary and angular; *dsa*, suture between dentary and surangular; *gl*, glenoid; *lf*, lacrimal facet; *lsa*, left surangular; *mxf*, maxillary facet; *pop*, postorbital process of jugal; *q*, quadrate; *qj*, quadratojugal; *qjp*, quadratojugal process of jugal; *rsa*, right surangular; *sa*, surangular; *saf*, surangular foramen; *sas*, suture between surangular and angular. Scale bars equal 10 cm.

The left and right surangulars and left angular are well preserved. The left surangular and angular articulate with the caudal end of the dentary along an almost vertical suture (Fig. 23A). The surangular slopes caudoventrally from the coronoid process towards the glenoid fossa for reception of the ventral condyle of the quadrate. The glenoid is a mediolaterally broad, cup-like depression with raised lateral and medial rims (Fig. 23A). A small surangular foramen pierces the lateral surface of the surangular rostroventral to the glenoid. A broad shallow depression occupies much of the medial surface of the surangular (Fig. 23B), forming the caudal part of the inframandibular fossa into which *M*. *adductor mandibulae posterior* probably inserted [28]. The surangular contacts the angular along a rostrocaudally elongate and inclined suture; the angular is fully visible in lateral view (Fig. 23A).

**Figure 24:**
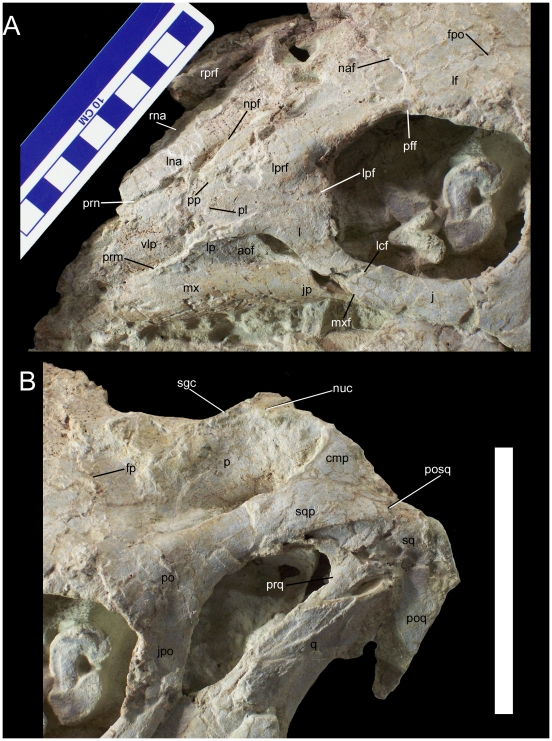
Skull of *Hippodraco scutodens*. (A) Left rostral region of the skull of UMNH VP 20208 in lateral view. (B) Left caudodorsal region of the skull in lateral view. *Abbreviations*: *aof*, antorbital fossa; *cmp*, caudomedial process of squamosal; *fp*, suture between left frontal and parietal; *fpo*, suture between left frontal and postorbital; *j*, jugal; *jp*, jugal process of maxilla; *jpo*; jugal process of postorbital; *l*, lacrimal; *lcf*, lacrimal facet; *lf*, left frontal; *lna*, left nasal; *lp*, lacrimal process of maxilla; *lpf*, suture between lacrimal and left prefrontal; *lprf*, left prefrontal; *mx*, maxilla; *mxf*, maxillary facet; *naf*, suture between left nasal and frontal; *npf*, suture between left nasal and prefrontal; *nuc*, nuchal crest; *p*, parietal; *pff*, suture between left prefrontal and frontal; *pl*, suture between premaxilla and lacrimal; *po*, postorbital; *poq*, postquadrate process; *posq*, caudal-most point on overlapping contact between postorbital and squamosal; *pp*, suture between premaxilla and left prefrontal; *prm*, suture between premaxilla and maxilla; *prn*, suture between premaxilla and left nasal; *prq*, prequadrate process; *q*, quadrate; *rna*, right nasal; *rprf*, right prefrontal; *sgc*, sagittal crest; *sq*, squamosal; *sqp*, squamosal process of postorbital; *vlp*, ventrolateral process of left premaxilla. Scale bars equal 10 cm.

The right premaxilla is missing, as are the rostral regions of both nasals and the left premaxilla. The ventrolateral process of the left premaxilla contacts the left maxilla along a roughly straight, rostroventrally to caudodorsally inclined suture along the former's ventral margin (Fig. 24A), as in *Theiophytalia kerri* (YPM 1887) and *Dakotadon lakotaensis* (SDSM 8656). Along its dorsal margin, the ventrolateral process contacts the left nasal. The ventrolateral process tapers at its caudal end, where it contacts the prefrontal caudodorsally and the lacrimal caudoventrally.

The left and right nasals meet along a midline suture on the dorsal surface of the skull (Fig. 24A). Both nasals are broken caudal to their premaxillary processes. The sutural relationships are best demonstrated by the more complete left nasal, which contacts along its lateral margin the ventrolateral process of the left premaxilla and the left prefrontal, and along its caudal margin meets the left frontal.

The left maxilla is nearly complete and well exposed in lateral view (Fig. 24A). It has been slightly displaced medially, such that it is no longer in full contact with the lacrimal and jugal along the respective contact surfaces. The lateral surface of the maxilla is strongly convex, resulting in a pronounced medial offset of the maxillary tooth row to correspond with the similarly offset tooth row of the dentary. The rostral margin of the maxilla is incomplete, thus rendering it difficult to assess the direction of the rostroventral process and the presence or absence of the rostrodorsal process. The ventral margin of the maxillary tooth row is straight. The ascending process of the maxilla is rostrocaudally broad and subtriangular in lateral view. A finger-like tab of bone, the lacrimal process, extends dorsally from the apex of the ascending process to contact the rostrodorsal process of the lacrimal (Fig. 24A). Caudoventral to the lacrimal process, the lateral surface of the maxilla becomes shallowly concave to form a rostrocaudally elongate elliptical depression, the antorbital fossa. Caudal to the fossa, the caudal margin of the maxillary ascending process is concave, forming the rostral edge of the antorbital fenestra. Ventral to the antorbital fossa, the lateral surface of the maxilla curves caudolaterally to form the jugal process (Fig. 24A). The jugal process is a sinuous ledge for reception of the maxillary process of the jugal, as in *Dakotadon lakotaensis* (SDSM 8656) and *Iguanacolossus fortis*. The maxillary teeth are obscured by matrix that would be very difficult to remove without endangering the surrounding bone, and thus their number and morphology are unknown; the only feature of the maxillary dentition that can be gleaned from the specimen is that only one active tooth in each tooth position participated in the occlusal plane.

The lacrimal is very similar in shape to those of *Dakotadon* (SDSM 8656) and *Theiophytalia* (YPM 1887). The rostrodorsal process of the lacrimal is dorsoventrally expanded and rounded along its rostral margin as in *Theiophytalia* and in more derived basal iguanodonts such as *Iguanodon bernissartensis* (IRSNB 1536); this process contacts the lacrimal process of the maxilla ventrally, the ventrolateral process of the premaxilla rostrally, and the prefrontal dorsally (Fig. 24A). The rostroventral margin of the lacrimal is concave to form the dorsal and caudal margins of the antorbital fenestra. The ventral ramus of the lacrimal tapers to a point and fits into a facet on the dorsal margin of the maxillary process of the jugal (Fig. 24A).

The left and right prefrontals are present, though the left is better preserved. The prefrontal is broad and flat at its rostral margin, contacting the rostrodorsal process of the lacrimal and ventrolateral process of the premaxilla ventrally and the nasal medially. The prefrontal becomes dorsoventrally narrower and its lateral surface more convex towards the caudal contact with the frontal (Fig. 24A). The orbital margin of the prefrontal is rugose.

The left frontal is better preserved than the right. The visible sutures between the left prefrontal and the left frontal, and between the left frontal and left postorbital, indicate that the frontal participated in the dorsal margin of the orbit (Figs. 20B, 24A). The orbital margin of the frontal is rugose. The dorsal surface of the frontal is flat. The left and right frontals meet along a midline suture and contact the parietal along an elongate transverse suture.

The parietal is broad and flat at its rostral margin where it contacts the frontals and postorbitals (Figs. 20B, 24B). Caudal to its contacts with the frontals and postorbitals, the parietal is highly constricted at its midpoint to form the medial margin of the supratemporal fenestra. The sagittal crest arises as a sharp ridge at approximately the midpoint of the parietal and extends caudally until it merges with the transversely broad nuchal crest (Fig. 24B). The parietal expands laterally along its caudal margin to contact the caudomedial process of the squamosal.

Only a fragment of the right postorbital remains, while the left is intact. The postorbital has a broad rostral contact with the frontal and a medial contact with the parietal; together with the suture between the frontal and parietal, these two contacts comprise a triradiate suture pattern on the skull roof (Fig. 20B). As with the prefrontal and frontal, the orbital margin of the postorbital is rugose, though the lateral surface of the postorbital is smooth. The jugal process of the postorbital protrudes rostroventrally to contact the postorbital process of the jugal. The squamosal process of the postorbital curves caudodorsally to meet the postorbital process of the squamosal; together, these two processes comprise the lateral margin of the supratemporal fenestra and the dorsal margin of the infratemporal fenestra (Figs. 20B, 24B). The squamosal process of the postorbital is rounded at its caudal extremity. The squamosal process of the postorbital extensively overlaps the postorbital process of the squamosal, reaching a point on the lateral surface of the squamosal directly dorsal to the glenoid fossa (Fig. 24B).

The left squamosal is well preserved, while the right is entirely missing. Much of the postorbital process of the squamosal is obscured by the aforementioned overlapping squamosal process of the postorbital. On the ventral surface of the squamosal, the glenoid fossa receives the dorsal end of the quadrate. The prequadrate process is a tapering prong that originates rostral to the glenoid and projects rostroventrally along the rostral margin of the jugal wing of the quadrate (Fig. 24B). The postquadrate process arises caudal to the glenoid and projects ventrally along the caudal margin of the quadrate. The postquadrate process tapers like the prequadrate process but is much broader in lateral view (Fig. 24B).

The left jugal is an elongate, strap-like bone. The maxillary process of the jugal forms the ventral margin of the orbit, with a concave dorsal margin and convex ventral margin (Fig. 23A). The rostral end of the maxillary process tapers to a point and contacts the jugal process of the maxilla along a rostroventral facet and the ventral ramus of the lacrimal along a rostrodorsal facet. The postorbital process of the jugal projects dorsally to meet the jugal process of the postorbital. The quadratojugal process of the jugal is broader than the postorbital process in lateral view (Fig. 23A). The ventral margin of the jugal is sinuous in lateral view, being convex ventral to the orbit, concave ventral to the postorbital process, and convex ventral to the infratemporal fenestra and quadratojugal process. The ventral margin of the jugal terminates in a caudoventrally directed, finely striated flange that projects caudal to the quadratojugal contact (Fig. 23A), as in *Theiophytalia kerri* (YPM 1887).

Only the left quadratojugal and quadrate are preserved, and these have suffered some rostral displacement (Fig. 23A). Due to fragmentation of the bone surface in this area, it is difficult to discern the contact between the quadratojugal and the quadrate (Fig. 20B), though it is clear that the quadratojugal overlapped the lateral surface of the jugal wing of the quadrate to some extent and that there was no gap (paraquadrate foramen) between the quadratojugal and the quadrate as is the case in *Theiophytalia*. The quadrate curves caudally along its entire length. The dorsal condyle of the quadrate is still articulated within the glenoid fossa of the left squamosal. The ventral condyle is slightly damaged but was clearly broader mediolaterally than it was long rostrocaudally.

**Figure 25:**
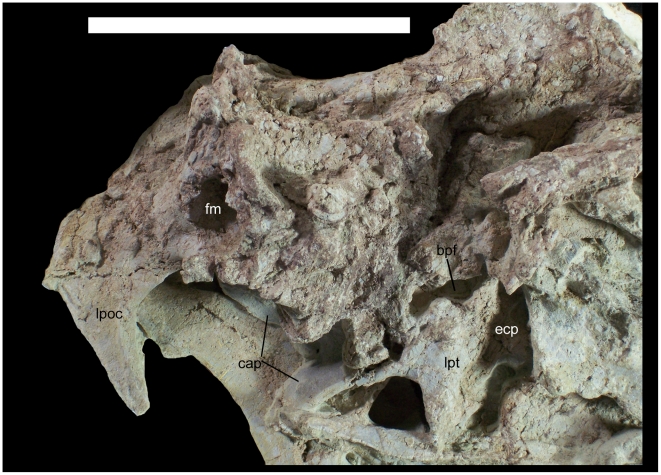
Skull of *Hippodraco scutodens*. Left pterygoid of UMNH VP 20208 in medial view and braincase of UMNH VP 20208 in caudal view. *Abbreviations*: *bpf*, basipterygoid process facet; *cap*, caudal alar process; *ecp*, ectopterygoid process; *fm*, foramen magnum; *lpoc*, left paroccipital process; *lpt*, left pterygoid. Scale bar equals 10 cm.

The medial side of the skull does not reveal a great deal of additional information on the cranial bones; though individual bones can be discerned, fragmentation of the bone surfaces and overlap of several elements make interpreting the medial side very difficult and somewhat speculative. Most of the precise sutural relationships on the medial side are nebulous (Fig. 20D). The left pterygoid is visible dorsal to the left surangular (Fig. 25). The ectopterygoid process is broad dorsoventrally with a wing-like projection along its ventral margin; the contacts with the ectopterygoid and maxilla are not visible. There is a marked depression on the dorsomedial surface of the pterygoid that likely represents the contact surface for the left basipterygoid process of the basisphenoid. The caudal alar process projects caudally towards the pterygoid wing of the left quadrate and bifurcates at its contact with the pterygoid wing, with one branch projecting caudoventrally and the other caudodorsally (Figs. 20D, 25).

The braincase itself is poorly preserved, with the ventral and right lateral surfaces severely damaged; the caudal aspect is more intact, although the nature of the sutures between the exoccipitals and supraoccipital cannot be determined, e.g., whether the latter is excluded from the foramen magnum. The left paroccipital process is complete; it is pendant and at its base projects caudolaterally relative to the braincase. The paroccipital process projects ventrally at its distal end (Fig. 25).

Most of the postcranial elements preserved in UMNH VP 20208 were collected in a large jacket with the skull; many of these elements have been fully freed from the matrix, although some vertebrae (at least an additional cervical and two dorsals) and dorsal ribs remain jumbled together to such a degree that separating them would be almost impossible without damaging them. The elements described in the following paragraphs are those that have been freed from the jacket.

**Figure 26:**
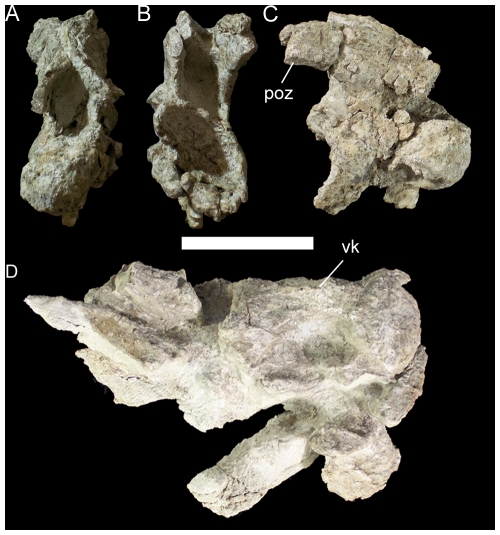
Cervical vertebrae of UMNH VP 20208. Cranial cervical vertebra of UMNH VP 20208 in (A) cranial, (B) caudal, and (C) right lateral views. (D) Two articulated cervical centra in ventral view. *Abbreviations*: *poz*, postzygapophysis; *vk*, ventral keel. Scale bar equals 5 cm.

The approximate positions of the preserved three cervical and three dorsal vertebrae have been inferred from basal iguanodonts for which complete, articulated cervical and dorsal series are known, including “*Camptosaurus*” *aphanoecetes* (CM 11337) [29], *Iguanodon bernissartensis* (IRSNB 1534) [30], and *Mantellisaurus atherfieldensis* (IRSNB 1551) [36]. Of the cervical series there is a nearly complete cranial cervical (possibly cervical 3 or 4) and two articulated centra. The cranial cervical has suffered mediolateral distortion but nevertheless is clearly strongly opisthocoelous, with a bulbous convex cranial face and concave, cup-like caudal face (Fig. 26A, B). The parapophyses cannot be distinguished due to fracturing. The prezygapophyses are missing as well. The postzygapophyses extend caudolaterally and curve caudodorsally from the apex of the neural arch (Fig. 26C). The two articulated cervical centra have suffered dorsoventral compression, but nevertheless the more cranial of the two displays a prominent keel along its ventral margin (Fig. 26D).

**Figure 27:**
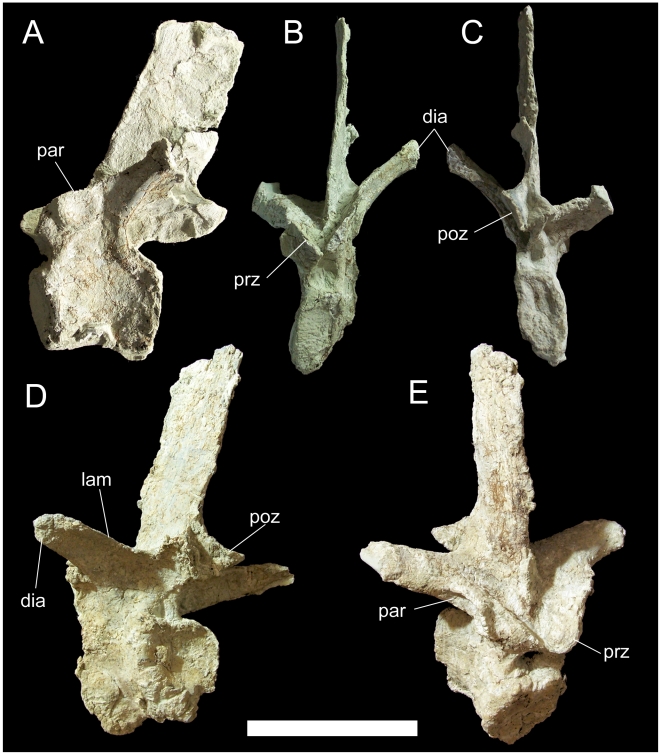
Dorsal vertebrae of UMNH VP 20208. Middle dorsal vertebra of UMNH VP 20208 in (A) left lateral, (B) cranial, and (C) caudal views. Caudal dorsal vertebra in (D) oblique caudolateral and (E) oblique craniodorsal views. *Abbreviations*: *dia*, diapophysis; *lam*, lamina; *par*, parapophysis; *poz*, postzygapophysis; *prz*, prezygapophysis. Scale bar equals 10 cm.

One of the dorsals is from the middle of the series, probably from between dorsals 6 and 11, based upon the direction of the transverse processes, inclination of the neural spine, and the size and position of the parapophyses. The centrum is slightly opisthocoelous, with a flat cranial face and shallowly concave caudal face. The centrum is hourglass-shaped in lateral view, with a bowed ventral margin (Fig. 27A). The centrum is much taller dorsoventrally and than it is wide mediolaterally, although some distortion has occurred. The transverse processes project dorsolaterally from the neural arch and terminate in flat, somewhat rugose diapophyses. The parapophyses are shallow depressions with raised rims immediately cranial to the base of the transverse processes (Fig 27A.). The prezygapophyses are cranially-projecting flanges dorsal to the parapophyses; the flat articular surfaces are directed dorsomedially (Fig. 27B). The postzygapophyses arise from the caudal margin of the neural spine and have articular surfaces that face ventrolaterally (Fig. 27C). The neural spine is inclined caudally and is almost perfectly rectangular in lateral view (Fig. 27A).

**Figure 28:**
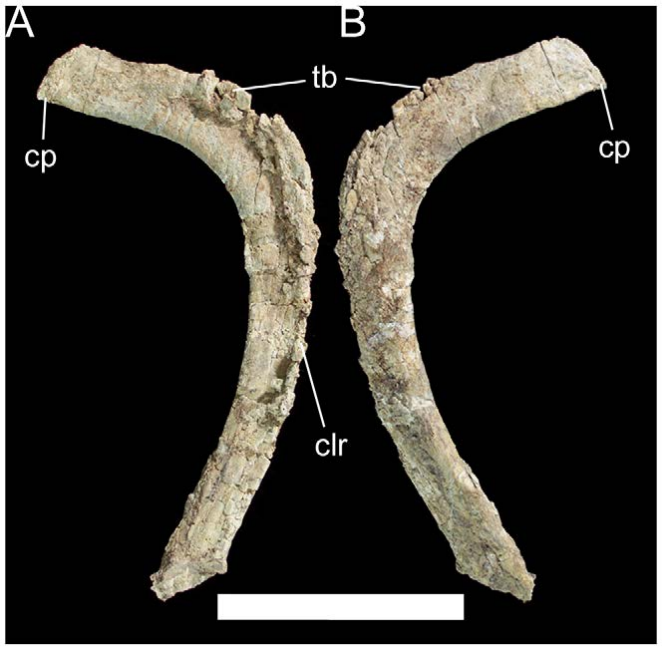
Left dorsal rib of UMNH VP 20208. Shown in (A) cranial and (B) caudal views. *Abbreviations*: *cp*, capitulum; *clr*, craniolateral ridge; *tb*, tuberculum. Scale bar equals 10 cm.

The other two dorsals are probably from near the caudal end of the dorsal series, as indicated by parapophyses situated on the transverse processes. One of these dorsals is overall rather poorly preserved, with severe fracturing and distortion such that the ventral surface of the centrum is fully visible in caudal view. However, the other caudal dorsal is nearly intact and allows full description of a representative caudal dorsal of *Hippodraco*. The centrum is similar to that of the middle dorsal described above. The bases of the transverse processes are craniocaudally elongate in dorsal view. The transverse processes project dorsolaterally, have a sharp lamina along their caudal margins, and end in the diapophyses (Fig. 27D). The parapophyses are cup-like depressions on the craniolateral margins of the transverse processes (Fig. 27E). The prezygapophyses are two rounded tabs with flat, dorsomedially-directed articular faces situated cranial to the bases of the transverse processes. The postzygapophyses are similar to those of the middle dorsal, arising from the caudal margin of the neural spine and with ventrolaterally-directed articular faces. The neural spine is inclined caudally. The dorsal ribs are typical of basal iguanodonts, with an elongate rectangular capitulum and a small rugose tuberculum (Fig. 28). The cranial surface of the rib shaft is shallowly concave with a ridge along the rostrolateral margin; the caudal surface is gently convex.

**Figure 29:**
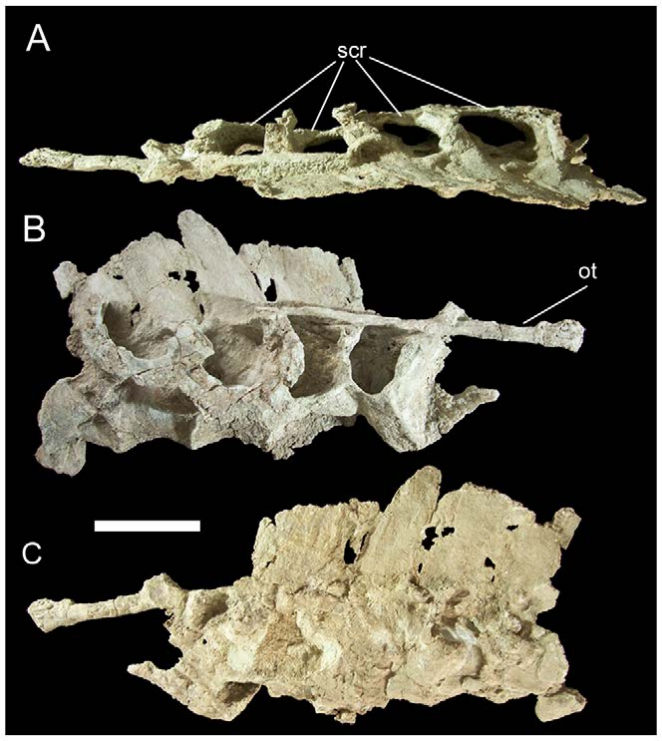
Partial sacrum of UMNH VP 20208. Shown in (A) dorsal, (B) right lateral, and (C) left lateral views. *Abbreviations*: *ot*, ossified tendons; *scr*, sacral ribs. Scale bar equals 10 cm.

UMNH VP 20208 includes a partial sacrum consisting of four co-ossified vertebrae. The centra of these sacral vertebrae have been mediolaterally compressed, obscuring their true shape and rendering it difficult to determine which sacrals are present. The right transverse processes are present and project laterally; they are connected to each other via the preserved right sacral ribs that would have articulated with the sacral rib facets of the right ilium (Fig. 29A). A bundle of ossified tendons extends horizontally along the right lateral sides of the bases of the neural spines (Fig. 29B). The neural spines are caudally inclined (Fig. 29B, C).

**Figure 30:**
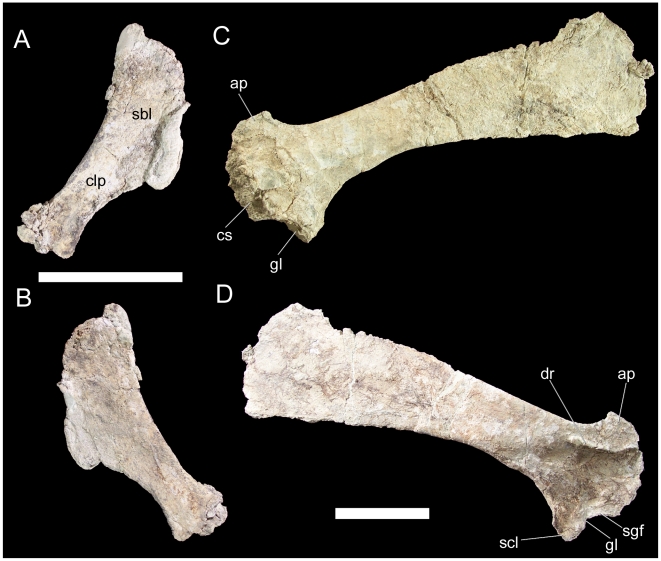
Pectoral elements of UMNH VP 20208. Left sternal of UMNH VP 20208 in (A) dorsal and (B) ventral views. Right scapula of UMNH VP 20208 in (C) medial and (D) lateral views. *Abbreviations*: *ap*, acromion process; *clp*, caudolateral process; *cs*, corocoid suture; *dr*, deltoid ridge; *gl*, glenoid; *sbl*, sternal blade; *scl*, scapular labrum; *sgf*, supraglenoid fossa. Scale bars equal 10 cm.

The left sternal is complete and uncrushed. The dorsal surface is slightly concave while the ventral surface is flat. The overall shape of the sternal resembles that of a hatchet, with a transversely broad kidney-shaped blade and narrower caudolateral process (Fig. 30A, B). The medial margin of the blade of the sternal is gently convex and rugose where it would have met the corresponding margin of the right sternal, while the lateral margin is concave and smooth. This concave lateral margin of the blade curves smoothly into the concave lateral margin of the caudolateral process. The concave medial margin of the caudolateral process forms a sharp angle with the convex medial margin of the blade (Fig. 30A, B). The caudal end of the caudolateral process is transversely expanded.

The right scapula is also complete and well preserved, although the margins are somewhat abraded. The cranial end of the scapula is mediolaterally thickened along the convex sutural surface for the coracoid (Fig. 30C); the abraded surface here makes it difficult to discern the canal that would have formed the caudal margin of the coracoid foramen. The scapular portion of the glenoid is a cranioventrally-facing concavity, bounded caudally by a ventrally directed subtriangular spur, the scapular labrum (Fig. 30D). Dorsal to the glenoid is a slight semi-circular concavity, the supraglenoid fossa. Dorsal to the supraglenoid fossa, a low shelf, the deltoid ridge, extends along the lateral surface of the scapula; the ridge extends caudoventrally from the cranial margin of the base of the acromion process and continues caudally ventral to the acromion process (Fig. 30D). The deltoid ridge becomes steadily thicker dorsoventrally towards its caudal end. The acromion process is a roughly subrectangular dorsally-projecting flange on the dorsal margin of the cranial end of the scapula; the craniodorsal margin of the process is convex, while the caudal margin is concave (Fig. 30D). Caudal to the deltoid ridge, the scapula immediately becomes much narrower mediolaterally to form the scapular blade. The gently convex dorsal margin and concave ventral margin diverge caudally to form the expanded, paddle-shaped caudal end of the scapula (Fig. 30C, D).

**Figure 31:**
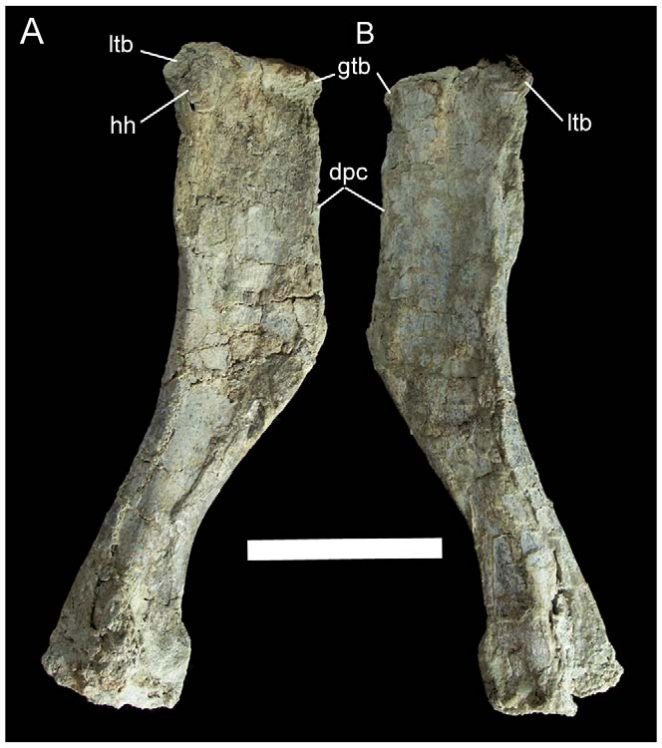
Right humerus of UMNH VP 20208. Shown in (A) caudal and (B) cranial views. *Abbreviations*: *dpc*, deltopectoral crest; *gtb*, greater tuberosity; *hh*, humeral head; *ltb*, lesser tuberosity. Scale bar equals 10 cm.

The right humerus has suffered some damage to the radial and ulnar condyles but is otherwise intact. The proximal end is subrectangular and rugose along its proximal edge with a prominent, roughly circular humeral head restricted to the caudal aspect of the proximal end (Fig. 31A). The greater and lesser tuberosities are rugose ledges lateral and medial to the humeral head, respectively. The deltopectoral crest extends from just distal to the greater tuberosity to a point less than halfway down the humeral shaft; at its distal margin, the crest merges gently with the lateral margin of the humeral shaft (Fig. 31A, B). The shaft of the humerus curves medially towards its distal end.

**Figure 32:**
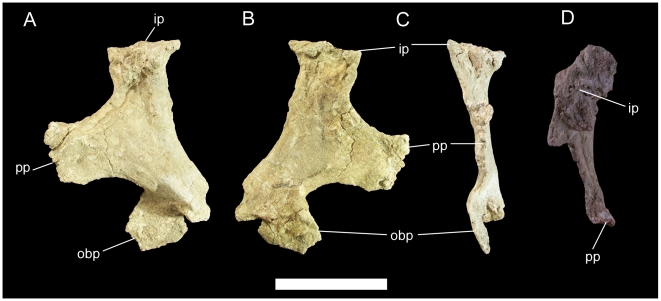
Left ischium of UMNH VP 20208. Shown in (A) lateral, (B) medial, (C) cranial, and (D) dorsal views. *Abbreviations*: *ip*, iliac peduncle; *obp*, obturator process; *pp*, pubic peduncle. Scale bar equals 10 cm.

The proximal portion of the left ischium is present; the ischial shaft is broken just distal to the obturator process (Fig. 32A, B). The obturator process is a tab of bone projecting cranioventrally from the craniomedial margin of the ischial shaft. The pubic peduncle is transversely compressed; the sutural surface for the ischial peduncle of the left pubis is rugose and thickens towards its dorsal margin (Fig. 32C). The iliac peduncle is considerably more robust, with a sutural surface that is roughly subrectangular in shape (Fig. 32D).

**Figure 33:**
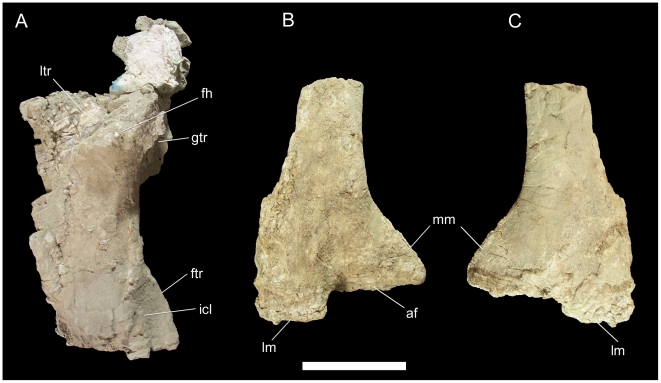
Femur and tibia of UMNH VP 20208. Right femur of UMNH VP 20208 in (A) medial view. Right tibia in (B) cranial and (C) caudal views. *Abbreviations*: *af*, facet for the astragalus; *fh*, femoral head; *ftr*, fourth trochanter; *gtr*, greater trochanter; *icl*, insertion site of *M. caudifemoralis longus*; *lm*, lateral malleolus; *ltr*, lesser trochanter; *mm*, medial malleolus. Scale bar equals 10 cm.

The proximal half of the right femur is preserved. The lateral surface is badly fractured and much of it obscured by matrix and an agglomeration of bone fragments. The medial surface, in contrast, does present some features for description. The femoral head is severely abraded and so its shape cannot be discerned. The badly fragmented lesser and greater trochanters are visible respectively craniolateral and caudolateral to the femoral head (Fig. 33A). Distal to the head, the preserved portion of the femoral shaft is straight. Part of the fourth trochanter projects caudally from the caudomedial margin of the femoral shaft; the shape of this incomplete trochanter is uncertain. Along the medial surface of the fourth trochanter and extending onto the cranial surface of the femur is a shallow depression that forms the presumed insertion site for *M*. *caudifemoralis longus* (Fig. 33A).

The distal end of the right tibia is present as well. The preserved portion of the tibial shaft is straight. The rectangular lateral malleolus is set off from the larger, triangular medial malleolus by a pronounced step (Fig. 33B, C). The bone surface of the lateral malleolus is badly fragmented, obscuring the articulation surface for the right calcaneum. The caudodistal surface of the medial malleolus bears a shallow facet for the articulation of the right astragalus (Fig. 33B).

**Figure 34:**
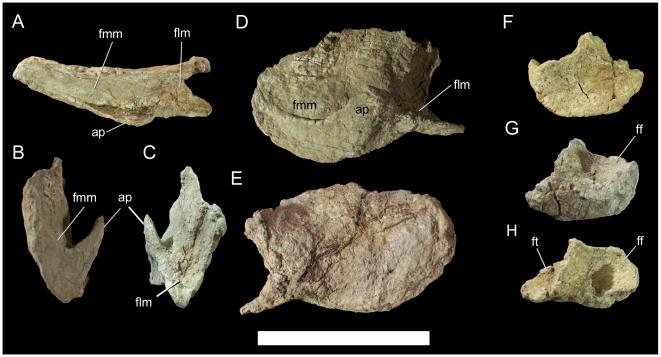
Tarsal elements of UMNH VP 20208. Left astragalus of UMNH VP 20208 in (A) proximal, (B) medial, (C) lateral, (D) cranial, and (E) caudal views. Left calcaneum in (F) lateral, (G) medial, and (H) proximal views. *Abbreviations*: *ap*, ascending process; *ff*, facet for fibula; *flm*, facet for lateral malleolus; *fmm*, facet for medial malleolus; *ft*, facet for tibia. Scale bar equals 10 cm.

The left astragalus and calcaneum are nearly intact. The concave proximal surface of the astragalus is traversed by a low craniocaudally oriented ridge, which divides the proximal surface into two facets to accommodate the lateral and medial malleoli of the tibia (Fig. 34A). The facet for the medial malleolus is mediolaterally elongate and craniocaudally narrow, corresponding to the astragalar articulation surface on the medial malleolus of the tibia (Fig. 34B). The facet for the lateral malleolus is incomplete but would likely have formed a cupped depression for articulation with the lateral malleolus of the tibia. There is a considerable offset between the facets for the lateral and medial malleoli; the facet for the lateral malleolus is situated more ventrally on the astragalus (Fig. 34B, C, D). The triangular ascending process arises from the cranial margin of the proximal surface (Fig. 34D). The caudal surface of the astragalus is broad, oval, and gently convex (Fig. 34E).

The calcaneum is shallowly concave on its lateral surface; this concavity is bounded by a raised rim (Fig. 34F). The dorsal surface of the calcaneum is divided by a sharp dorsally-projecting ridge into two depressions (Fig. 34G, H). The larger, circular, and more cranial depression is a facet for the articulation of the distal end of the fibula, while the smaller, elliptical, and more caudal depression would have articulated with the craniodistal surface of the lateral malleolus of the tibia [30], [36]. The ventral margin of the calcaneum is broadly convex.

**Figure 35:**
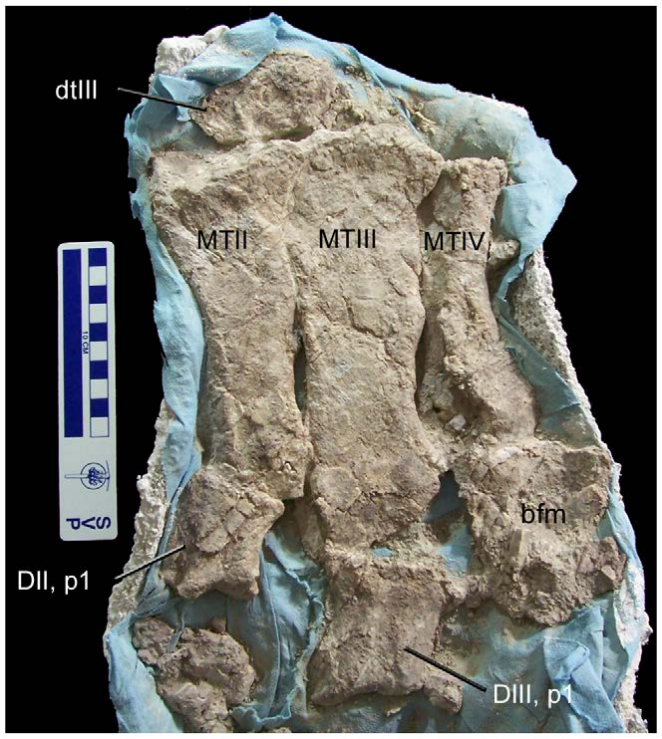
Left metatarsus of UMNH VP 20208. Shown in dorsal view. *Abbreviations*: *bfm*, bone fragments and matrix; *DII*, *p1*, phalanx 1 of digit II; *DIII*, *p1*, phalanx 1 of digit III; *dtIII*, distal tarsal III; *MTII–IV*, metatarsals II, III, and IV. Scale bar equals 10 cm.

The left pes is complete except for digit I and bears a close resemblance to those of *Camptosaurus dispar* (YPM 1877) and *Iguanodon bernissartensis* (IRSNB 1534), which are used herein to interpret the pes of UMNH VP 20208. A flat, oval-shaped bone adjacent to the proximal ends of MTII and III is likely distal tarsal III (Fig. 35). The proximal end of MTII overlaps that of MTIII with a tab of bone on the craniomedial margin. The shaft of MTII curves gently medially towards its distal end. MTIII is straight and overlaps the proximal end of MTIV. MTIV is strongly curved laterally and is the shortest of the three preserved metatarsals (Fig. 35). The presence or absence of MTI, and thus digit I, cannot be assessed.

**Figure 36:**
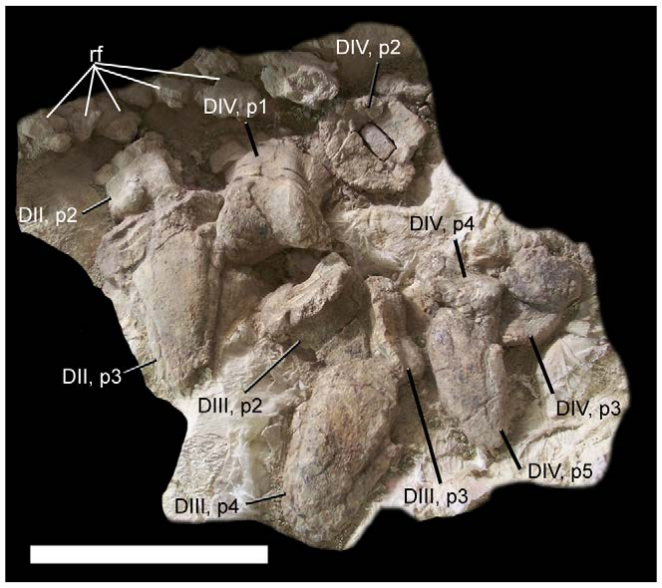
Left pes of UMNH VP 20208. Shown in dorsal view. *Abbreviations*: *D*, digit; *p*, phalanx; *rf*, rock fragments. Scale bar equals 10 cm.

The left pedal phalanges are all present, well preserved, and semi-articulated (Fig. 36). The identifications put forth in Figure 36 are based upon comparison with YPM 1877 and IRSNB 1534. As in these specimens, the three proximal pedal phalanges of UMNH VP 20208 are the largest of their respective digits, with an elongate rectangular shape. More distal phalanges become progressively shorter proximodistally before culminating in the pedal unguals. Phalanx 2 of digit III exhibits a prominent lip on its proximodorsal margin, similar to the distal phalanges of YPM 1877 and IRSNB 1534. The unguals are elongate and claw-like, but with blunt, rounded tips, like those of IRSNB 1534. There are sulci on the lateral and medial surfaces of the unguals. The expected phalangeal formula for pedal digits II, III, and IV is 3-4-5.

### Other Ornithopods from Andrew's Site

**Figure 37:**
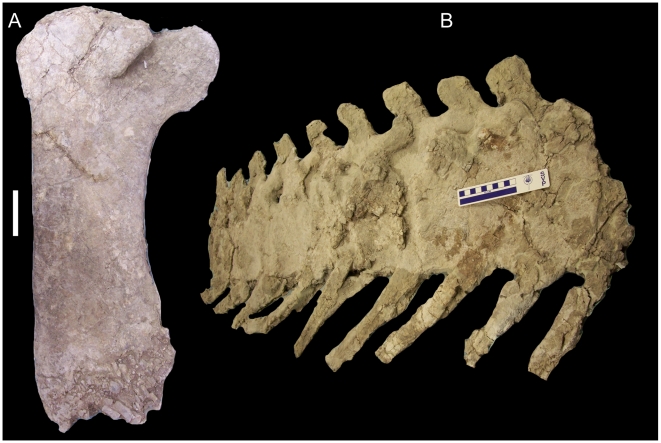
UMNH VP 20207. Partial left tibia (A, lateral view) and proximal caudal vertebrae and chevrons (B, right craniolateral view) of an indeterminate large iguanodont from Andrew's Site, upper Yellow Cat Member. Scale bars equal 10 cm.

In addition to UMNH VP 20208, the holotype of *Hippodraco scutodens*, two other ornithopods were excavated from the locality known as Andrew's Site. UMNH VP 20207 includes the proximal left tibia, an indeterminate additional appendicular element, and nine articulated proximal caudal vertebrae and chevrons of an extremely large iguanodont (Fig. 37). Unfortunately, there are no overlapping elements to elucidate whether UMNH VP 20207 pertains to a larger individual of *Hippodraco*, and there are no preserved features shared with any other iguanodont from the Cedar Mountain Formation that would allow a definite referral of the specimen; UMNH VP 20207 should therefore be considered an indeterminate iguanodontian.

**Figure 38:**
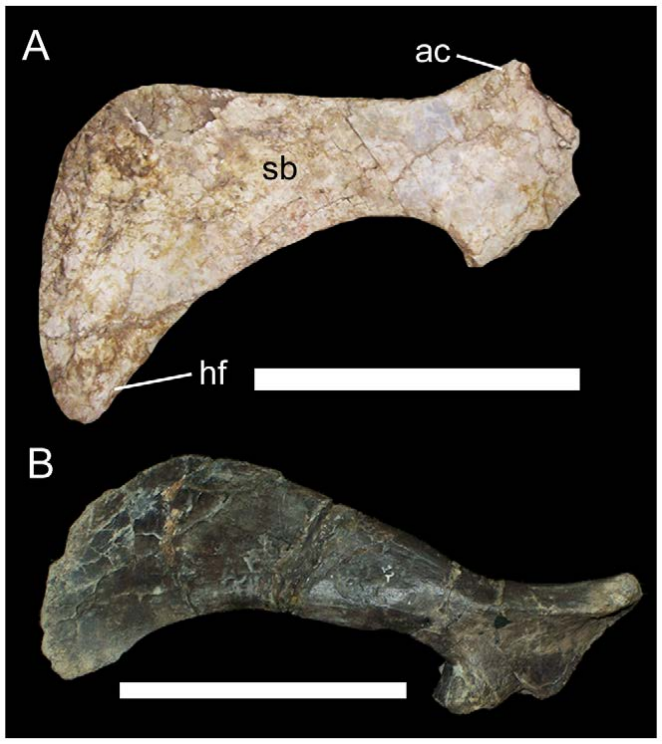
UMNH VP 20644. Right scapula of a basal ornithopod from Andrew's Site, upper Yellow Cat Member, in (A) lateral view. Right scapula of NHMUK R196, a partial of skeleton of *Hypsilophodon foxii*, in (B) lateral view. *Abbreviations*: *ac*, acromion process; *hf*, hook-like flange; *sb*, scapular blade. Scale bar in A equals 10 cm; scale bar in B equals 5 cm.

UMNH VP 20644 is a small right scapula. The cranial end is fragmented, but the scapular blade is complete and quite different in form from that of *Hippodraco*. The dorsal margin of the blade is straight while the ventral margin is deeply concave and terminates in a very prominent hook-like flange (Fig. 38A). The shape of the scapular blade of UMNH VP 20644 is remarkably similar to that of the right scapula of NHMUK R196, a partial skeleton of *Hypsilophodon foxii* from the Isle of Wight (Fig. 38B), suggesting that UMNH VP 20644 is the first indication of a *Hypsilophodon*-grade basal ornithopod from the Cedar Mountain Formation. UMNH VP 20644 is best regarded as an indeterminate basal ornithopod.

## Discussion

**Figure 39:**
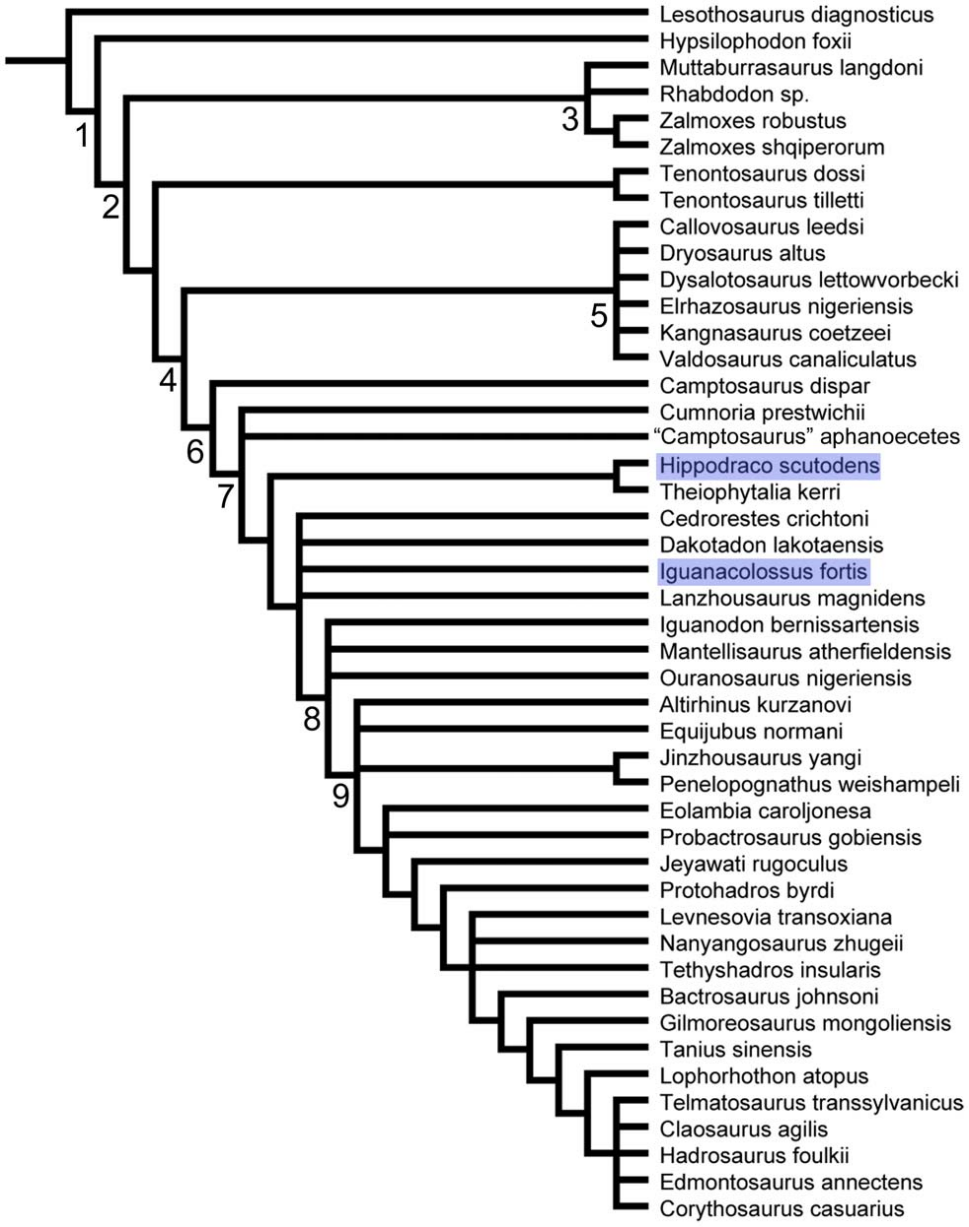
Phylogenetic relationships of *Iguanacolossus fortis* and *Hippodraco scutodens*. Reduced consensus tree of 11,850 MPTs of 358 steps each, following ordering of 22 multistate characters and safe taxonomic reduction of “*Camptosaurus*” *valdensis* and *Draconyx loureiroi*. The new taxa *Iguanacolossus fortis* and *Hippodraco scutodens* are highlighted. Numbers below and to the left of some nodes correspond to the following clade names: 1, Ornithopoda; 2, Iguanodontia; 3, Rhabdodontidae; 4, Dryomorpha; 5, Dryosauridae; 6, Ankylopollexia; 7, Styracosterna; 8, Hadrosauriformes; 9, Hadrosauroidea.

A new phylogenetic analysis of basal iguanodonts was performed to elucidate the relationships of *Hippodraco* and *Iguanacolossus*. The initial version of this analysis recently appeared in McDonald et al. [37]. The analysis has been updated in two important ways: “*Dollodon bampingi*” and *Mantellisaurus atherfieldensis* are now coded as a single taxon (*M*. *atherfieldensis* [ATM, in review]) and the number of replicates used in the search has been increased from 10 to 10,000. The analysis of 61 OTUs and 131 morphological characters was run in TNT [38]. The character list (File S1), data matrix (File S2), list of specimens examined (File S3), and bibliography of supplemental references (File S4) are available as online supplementary information. The starting trees were Wagner with a random seed of 1 and 10,000 replicates; the tree bisection reconnection algorithm was used with 10 trees saved per replication. The analysis was run with 22 multistate characters designated as ordered (additive in the terminology of TNT), with order determined by the method of intermediates [39]. This resulted in 13,080 MPTs of length 358 steps; the strict consensus cladogram suffered from an almost total lack of resolution, with Dryomorpha simply a massive polytomy of Dryosauridae (*Callovosaurus leedsi*, “*Camptosaurus*” *valdensis*, *Dryosaurus altus*, *Dysalotosaurus lettowvorbecki*, *Elrhazosaurus nigeriensis*, *Kangnasaurus coetzeei*, and *Valdosaurus canaliculatus*) plus all members of Ankylopollexia. To explore the possibility of improving resolution of the strict consensus tree through safe taxonomic reduction, the matrix was examined in the program TAXEQ3 [40] using a randomization test. This indicated that “*Camptosaurus*” *valdensis* and *Draconyx loureiroi* could be safely omitted in the second running of the analysis. The second running of the analysis following safe taxonomic reduction found 11,850 MPTs of 358 steps (CI  = 0.534, RI  = 0.840). The strict consensus tree resolved the base of Dryomorpha with a node uniting taxa more derived than Dryosauridae into Ankylopollexia; however, within Ankylopollexia, all taxa formed an unresolved polytomy. To achieve greater resolution, the matrix was run through the program REDCON 3.0 [41], which calculated 20 reduced consensus trees. Trees 2, 3, 6, 7, and 19 were combined to arrive at the tree shown in Figure 39, leading to the *a posteriori* deletion of 13 OTUs (*Owenodon hoggii*, NHMUK R8676, *Planicoxa venenica*, “*Camptosaurus*” *depressus*, NHMUK R1831, *Barilium dawsoni*, *Hypselospinus fittoni*, *Kukufeldia tilgatensis*, *Lurdusaurus arenatus*, *Fukuisaurus tetoriensis*, *Probactrosaurus mazongshanensis*, *Jintasaurus meniscus*, and *Shuangmiaosaurus gilmorei*). The following discussion is based upon this cladogram. *Hippodraco* and *Iguanacolossus* are both basal styracosternans. *Hippodraco scutodens* forms a small clade with *Theiophytalia kerri* from the Aptian–Albian Lytle Member of the Purgatoire Formation of Colorado [11], [42]. It is also necessary to note that the genus *Camptosaurus* is found to be paraphyletic in the reduced consensus tree, with “*Camptosaurus*” ( =  *Cumnoria*) *prestwichii* and “*C*.” *aphanoecetes* being more derived than *C*. *dispar*. The taxonomy of *Camptosaurus* will be addressed elsewhere [ATM, in review].

The novel phylogenetic analysis implies a complex paleobiogeographic history of Early Cretaceous basal iguanodonts. The inclusion of *Muttaburrasaurus* from the Albian of Australia [43] in Rhabdodontidae, the basal-most subclade in Iguanodontia and otherwise known only from the Campanian–Maastrichtian of Europe [44], [45], fills part of a considerable ghost lineage and suggests a far wider distribution of rhabdodonts than previously supposed. However, it is necessary to note that while material of the European rhabdodonts *Rhabdodon* and *Zalmoxes* has been examined firsthand by the lead author, only casts (NHMUK R9604) of several postcranial elements of *Muttaburrasaurus* have been examined; the actual holotype specimen (QM F6140), including skull material, has not yet been seen firsthand. Further study might well alter the phylogenetic placement of *Muttaburrasaurus*.

Another intriguing implication of the phylogenetic analysis and one particularly relevant to the new taxa from Utah is that basal iguanodonts from the Barremian–Aptian of western North America are more basal than contemporaneous taxa from Europe and Asia. For example, the upper Barremian–lowermost Aptian North American taxon *Hippodraco scutodens* is more basal than *Iguanodon bernissartensis* from the Sainte-Barbe Clays Formation of Belgium [30], [46] and *Jinzhousaurus yangi* from the Dakangpu Member of the Yixian Formation of China [2], [47]. Furthermore, the phylogenetic analysis suggests a degree of endemism among some basal iguanodonts in the Early Cretaceous. The clade of *Hippodraco* + *Theiophytalia* is known from only the Barremian–Albian of western North America, while *Jinzhousaurus* + *Penelopognathus* is known from only eastern Asia. The phylogeny and paleobiogeography of basal iguanodonts will be examined in more detail elsewhere [ATM, in preparation].

### Nomenclatural Acts

The electronic version of this document does not represent a published work according to the International Code of Zoological Nomenclature (ICZN), and hence the nomenclatural acts contained in the electronic version are not available under that Code from the electronic edition. Therefore, a separate edition of this document was produced by a method that assures numerous identical and durable copies, and those copies were simultaneously obtainable (from the publication date noted on the first page of this article) for the purpose of providing a public and permanent scientific record, in accordance with Article 8.1 of the Code. The separate print-only edition is available on request from PLoS by sending a request to PLoS ONE, 185 Berry Street, Suite 3100, San Francisco, CA 94107, USA along with a check for $10 (to cover printing and postage) payable to “Public Library of Science”.

In addition, this published work and the nomenclatural acts it contains have been registered in ZooBank, the proposed online registration system for the ICZN. The ZooBank LSIDs (Life Science Identifiers) can be resolved and the associated information viewed through any standard web browser by appending the LSID to the prefix “http://zoobank.org/”. The LSID for this publication is: urn:lsid:zoobank.org:pub:3E17C7FA-CB80-4D70-9398-DE509C450F3F.

## Supporting Information

File S1 Description of characters used in the phylogenetic analysis of Iguanodontia.
(0.07 MB DOC)

File S2 Data matrix used in the phylogenetic analysis of Iguanodontia.
(0.12 MB XLS)

File S3 List of the fossil specimens examined firsthand and references used to code the 61 taxa in the phylogenetic analysis of Iguanodontia.
(0.03 MB XLS)

File S4 List of the references cited in the character and specimen lists.
(0.05 MB DOC)
