# Supplementary material for: New Basal Iguanodonts from the Cedar Mountain Formation of Utah and the Evolution of Thumb-Spiked Dinosaurs
Source: PLoS One. 2010 Nov 22;5(11):e14075. doi: 10.1371/journal.pone.0014075 (PMC2989904; doi:10.1371/journal.pone.0014075)
Supplement: File S1 — Description of characters used in the phylogenetic analysis of Iguanodontia. (0.07 MB DOC) [file pone.0014075.s001.doc]

**Supplement 1.** Description of characters used in the phylogenetic analysis of Iguanodontia. Characters are numbered 0–130 in the format of TNT. Abbreviations refer to specific skeletal elements or regions (e.g., PD1 is predentary character 1): PD, predentary; DT, dentary; SU, surangular; ANG, angular; PM, premaxilla; MX, maxilla, LAC, lacrimal, PRF, prefrontal; PO, postorbital; JG, jugal; QJ, quadratojugal; QU, quadrate; SQ, squamosal; BC, braincase; T, teeth; AX, axial column; PC, pectoral girdle; FL, forelimb; PV, pelvic girdle; HL, hind limb. Characters are polarized with respect to the outgroup *Lesothosaurus diagnosticus*.

**Mandibular**

0. Predentary, overall shape of oral portion in dorsal view: subtriangular, comes to a point without a distinct rostral portion (0); arcuate, rounded anterolateral corners (1); subrectangular, squared corners and straight, well demarcated rostral portion (2) (modified from Weishampel *et al.,* 2003, character 18; and Prieto-Márquez *et al.,* 2006b, character 5). PD1 [ordered]

1. Predentary, directions of lateral margins of lateral processes relative to each other in dorsal and ventral views: divergent (0); parallel (1). PD2

2. Predentary, morphology of ventromedial process: undivided (0); bifurcated (1) (Weishampel *et al.,* 2003, character 20). PD3

3. Predentary, dorsomedial process: absent (0); present (1). PD4

4. Predentary, denticles: absent (0); present (1) (Weishampel *et al.,* 2003, character 19). PD5

5. Predentary, denticle morphology: large, conical median denticle with one or two prominent conical denticles of subequal size adjacent to the median denticle on both sides and smaller, tab-like denticles on lateral processes (0); rostrocaudally compressed prong-like denticles that increase in size towards the median denticle, which is largest (1); rostrocaudally compressed prong-like denticles of equal size (2) (modified from Prieto-Márquez 2010, characters 25 and 27) PD6 [ordered]

6. Predentary, grooves on either side of midline on rostral surface, extending ventrolaterally to dorsomedially: absent (0); present (1). PD7

7. Dentary, orientation of symphysis relative to lateral margin of dentary: rostrolateral to caudomedial (medial edge of symphysis and lateral margin of dentary diverge in dorsal view) (0); parallel (1) (Prieto-Márquez *et al.,* 2006b, character 10). DT1

8. Dentary, orientation of symphyseal region in lateral view: ventral surface of symphyseal region visible in lateral view, articulation site for ventromedial process of predentary faces ventolaterally (0); ventral surface of symphyseal region not visible in lateral view, articulation site for ventromedial process of predentary faces ventrally (1). DT2

9. Dentary, diastema: absent (0); present (1). DT3

10. Dentary, shape of tooth row in dorsal view: bowed medially at mid-length (0); bowed medially along caudal half (1); straight (2) (modified from Prieto-Márquez *et al.,* 2006b, character 8). DT4 [ordered]

11. Dentary, shape of tooth row in lateral view: straight (0); concave (1); convex (2). DT5

12. Dentary, orientation of tooth row relative to lateral surface of dentary: convergent rostrally and caudally (0); convergent rostrally and divergent caudally (1). DT6

13. Dentary, morphology of tooth alveoli: alveoli shaped by dentary teeth (0); alveoli with parallel vertical walls (1) (Norman 2002, character 33). DT7

14. Dentary, caudal-most extent of tooth row: rostral to base of coronoid process (0); medial to coronoid process but still rostral to longitudinal axis of the process (1); even with longitudinal axis of the coronoid process (2); caudal to longitudinal axis of the coronoid process but still rostral to the caudal margin of the process (3); caudal to the base of the coronoid process (4) (modified from You *et al.,* 2003b, character 29). DT8 [ordered]

15. Dentary, shape in lateral or medial view: tapers rostrally (0); dorsal and ventral margins are parallel (1); deepens rostrally (2) (Norman 2004, character 22). DT9

16. Dentary, morphology of ventral margin of rostral ramus leading to the predentary articulation: straight (0); ventral margin inflected ventrally, such that it curves gently towards the predentary articulation and symphysis (1); ventral margin curves dorsally towards symphysis (2). DT10

17. Dentary, bulge along ventral margin directly ventral to the base of the coronoid process: absent (0); present (1) (modified from Prieto-Márquez 2010, character 41). DT11

18. Dentary, bulge on the lateral surface ventral to the coronoid process that gives rise to the process: absent (0); present (1) (modified from Prieto-Márquez 2010, character 46). DT12

19. Dentary, platform between the tooth row and the coronoid process: absent, tooth row curves into base of coronoid process (0); present (1) (modified from Norman 2002, character 26). DT13

20. Dentary, orientation of coronoid process: caudally inclined (0); vertical (1); rostrally inclined (2) (modified from Prieto-Márquez *et al.,* 2006b, character 7). DT14 [ordered]

21. Dentary, expansion of dorsal end of coronoid process: absent (0); present (1). DT15

22. Dentary, expansion of dorsal end of coronoid process, location: along rostral edge only (0); along rostral and caudal edges (1) (modified from McDonald *et al.,* 2010, character 33). DT16

23. Dentary, position of greatest rostrocaudal width of expanded coronoid process: ventral to apex (0); at apex (1). DT17

24. Surangular, surangular foramen: present (0); absent (1) (modified from Weishampel *et al.,* 1993, character 27). SU1

25. Surangular, external mandibular fenestra: large, open fenestra between dentary, surangular, and angular (0) small foramen (“accessory foramen”) on surangular near suture with dentary (1) absent (2) (modified from Kobayashi and Azuma 2003, character 15). SU2 [ordered]

26. Surangular, shape of contact with angular in lateral view: inclined rostrodorsal to caudoventral (0); sinuous (1); horizontal (2). SU3

27. Angular, exposure in lateral view: present, groove on ventral margin of surangular for articulation with angular (0); absent, articulation with surangular occurs on the medial surface of that bone (1) (modified from Norman 2002, character 28). ANG1

**Cranial**

28. Premaxilla, morphology of rostral margin in dorsal view: premaxillae not transversely expanded, snout comes to a point (0); premaxillae laterally expanded, snout squared (1). PM1

29. Premaxilla, tooth alveoli: present (0); absent (1). PM2

30. Premaxilla, ventral inflection: absent, oral margin even with ventral margin of maxilla (0); present, oral margin projects farther ventrally than ventral margin of maxilla (1) (modified from Norman 2002, character 2). PM3

31. Premaxilla, morphology of rostrolateral corner of oral margin in lateral view: in contact with maxilla (0); free and gently curved (1); free and angular (2). PM4 [ordered]

32. Premaxilla, everted rim on lateral edge of oral margin: absent (0); present (1) (modified from Weishampel *et al.,* 1993, character 3). PM5

33. Premaxilla, denticles on oral margin: absent (0); present (1) (modified from Weishampel *et al.,* 2003, character 7). PM6

34. Premaxilla, denticle morphology: one large conical denticle adjacent to interpremaxillary suture on each premaxilla (0); two large, rostrocaudally elongate denticles on each premaxilla (1); four or more conical denticles of similar size on each premaxilla (2); three rostrally projecting denticles that decrease in size laterally (3). PM7 [ordered]

35. Premaxilla, morphology of caudal ramus of ventrolateral process: tapers to a point (0); dorsoventrally expanded (1) (modified from Prieto-Márquez 2010, character 71). PM8

36. Premaxilla, contact with lacrimal: absent (0); present (1) (Weishampel *et al.,* 2003, character 8). PM9

37. Premaxilla, contact with prefrontal: absent (0); present (1). PM10

38. External naris, position: confined to area immediately above oral margin of premaxilla (0); extends caudally so as to lie dorsal to maxilla (1) (modified from Weishampel *et al.,* 2003, character 2). NA1

39. Maxilla, rostrodorsal process: absent (0); present (1) (modified from Prieto-Márquez *et al.,* 2006b, character 17). MX1

40. Maxilla, direction of rostroventral process: rostrally directed (0); rostroventrally curved (1). MX2

41. Maxilla, ventral margin of tooth row in lateral view: straight (0); concave (1). MX3

42. Maxilla, shape in dorsal view: bowed medially (0); straight for most of length (1); bowed laterally (2). MX4

43. Maxilla, shape of tooth row in ventral view: medially bowed, with rostral and caudal ends curving laterally (0); bowed laterally (1); straight (2). MX5

44. Maxilla, shape of ascending process: rostrocaudally narrow and hook-like (0); rostrocaudally broad and subtriangular (1). MX6

45. Maxilla, jugal process morphology: dorsally concave, rostrodorsally to caudoventrally inclined shelf, scarf contact with jugal (0); sinuous shelf, scarf contact with jugal (1); caudolaterally projecting jugal process, “finger-in-recess” contact with jugal (2); mediolaterally broad, flat surface against which jugal abuts (3) (modified from Norman 2002, character 15). MX7

46. Maxilla, antorbital fossa, extent in lateral view: occupies most of lateral surface of ascending process (0); rostrocaudally elongate, elliptical depression restricted to caudal half of ascending process (1); small semicircular depression restricted to caudal margin of ascending process (2); antorbital fossa not visible in lateral view (3). MX8 [ordered]

47. Lacrimal, concave ventral margin to form part of antorbital fenestra: present (0); absent (1). LAC1

48. Lacrimal, morphology of rostrodorsal ramus: tapers to a point (0); blocky and angular (1); bifurcated (2); dorsoventrally expanded (3). LAC2

49. Lacrimal, morphology of ventral ramus: tapers to a point (0); blocky and angular (1); rounded (2). LAC3

50. Lacrimal, contact with nasal: present (0); absent (1) (Norman 2002, character 12). LAC4

51. Prefrontal, morphology of nasal process: tapering, finger-like projection (0); dorsoventrally broad, mediolaterally compressed plate plate (1). PRF1

52. Postorbital, shape of caudal end of squamosal process that overlaps the lateral surface of the squamosal: tapers to a point (0); rounded (1); bifurcated (2) (modified from Prieto-Márquez 2010, character 132). PO1

53. Jugal, articulation with ectopterygoid: present (0); absent (1) (Head 1998, character 6). JG1

54. Jugal, morphology of portion of maxillary process that overlaps maxilla: tapers at rostral ends of maxillary and lacrimal contacts, with slightly convex ventral margin and slightly concave dorsal margin (0); subrectangular with parallel dorsal and ventral margins (1); tapers with sinuous dorsal and ventral margins (2); dorsoventrally expanded (3); dorsoventrally expanded to form part of rostral margin of orbit (4) (modified from Norman 2002, character 14). JG2

55. Jugal, large neurovascular foramen at base of postorbital process on medial surface: absent (0); present (1). JG3

56. Jugal, shape of free ventral margin caudal to maxillary and ectopterygoid contacts: straight (0); sinuous, jugal dorsoventrally expanded ventral to infratemporal fenestra (1); sinuous with striated, caudally-directed flange that projects caudal to jugal-quadratojugal contact (2); angular, with prominent ventrally-directed flange ventral to infratemporal fenestra (3); dorsoventrally narrow and strap-like, with convex ventral margin and concave dorsal margin that are parallel (4) (modified from Norman 2002, character 16). JG4

57. Jugal, contribution of caudal ramus to ventral margin of infraorbital fenestra: partial, quadratojugal also forms part of margin (0); caudal ramus forms entire ventral margin of infraorbital fenestra (1) (Weishampel *et al.,* 2003, character 11). JG5

58. Quadratojugal, quadratojugal foramen: absent (0); present (1) (Weishampel *et al.,* 2003, character 17). QJ1

59. Quadrate, quadratojugal notch in lateral wing: absent (0); present (1) (McDonald *et al.,* 2010, character 16). QU1

60. Quadrate, shape of notch in lateral wing: semicircular (0); broad and crescentic (1) (modified from Prieto-Márquez *et al.,* 2006b, character 40). QU2

61. Quadrate, paraquadrate foramen: absent, caudal margin of quadratojugal contacts entire rostral margin of quadrate along the contact surface (0); present, gap between portion of caudal margin of quadratojugal and rostral margin of quadrate (1). QU3

62. Quadrate, overall shape in lateral or medial view: curved gently caudally along entire length (0); straight for much of dorsoventral length, curved caudally near dorsal end (1); straight (2). QU4

63. Quadrate, shape of dorsal condyle: subtriangular, broad rostral margin and tapers to a point caudally (0); subrectangular (1); D-shaped, broadest along lateral profile (2) QU5

64. Quadrate, shape of ventral condyle: rostrocaudally narrow and mediolaterally broad, with larger lateral condylar surface and lateral and medial condyles on same plane (0); asymmetrical with enlarged, more ventrally situated lateral condyle (1) QU6

65. Squamosal, morphology of postorbital process dorsal to *M. adductor mandibulae externus* origin site: gently convex (0); mediolaterally compressed and blade-like (1). SQ1

66. Squamosal, orientation of caudomedial process: curved rostromedially (0); curved caudomedially (1); straight and medially directed (2) (modified from Prieto-Márquez *et al.,* 2006b, character 45). SQ2

67. Squamosal, relationship of right and left squamosals on skull roof: widely separated by parietal (0); separated by only a narrow band of the parietal (1); in broad contact with each other (2) (Horner *et al.,* 2004, character 63). SQ3 [ordered]

68. Frontal, participation in dorsal orbital rim: present (0); absent (1) (Norman 2002, character 19). BC1

69. Supraoccipital, contribution to foramen magnum: present (0); absent, excluded by exoccipitals (1) (You *et al.,* 2003b, character 23). BC2

70. Supraoccipital, morphology of supraoccipital-exoccipital contact: straight suture that meets squamosal (0); ventrolateral corner of supraoccipital is inset into exoccipital so that supraoccipital is locked between exoccipitals (1) (Horner *et al.,* 2004, character 66). BC3

71. Supraoccipital, inclination of caudal surface: caudal surface rostrally inclined (0); caudal surface vertical (1) (modified from Horner *et al.,* 2004, character 65). BC4

72. Exoccipital-Opisthotic, paroccipital process shape: dorsoventrally expanded distally (0); pendant (1) (Weishampel *et al.,* 2003, character 13). BC5

73. Exoccipital-Opisthotic, paroccipital process orientation of pendant distal portion: straight and ventrally directed (0); curved rostrally (1) (Horner *et al.,* 2004, character 62). BC6

74. Basioccipital, orientation of occipital condyle: caudoventrally directed (0); caudally directed (1) (modified from Prieto-Márquez 2010, character 152). BC7

75. Basioccipital, rostrocaudally directed groove extending along ventral surface: absent (0); present (1). BC8

76. Basioccipital, rostrocaudally directed, sharply defined ridge between basal tubera: absent (0); present (1). BC9

77. Basisphenoid, surface between basipterygoid processes: smooth (0); transverse, sharply defined ridge between basipterygoid processes (1); ventrally directed prong between basipterygoid processes (2) (modified from Gates and Sampson 2007, characters 78 and 79). BC10

78. Basisphenoid, orientation of basipterygoid processes: ventolaterally directed and rostrally inclined (0); ventrolaterally directed and caudally inclined (1) (modified from Prieto-Márquez *et al.,* 2006b, character 83). BC11

79. Foramen magnum, composition of ventral margin: caudomedial surfaces of left and right exoccipitals and dorsal margin of basioccipital (0); left and right exoccipitals only (1) (modified from Weishampel *et al.,* 1993, character 24). BC12

**Dental**

80. Dentary teeth, intercrown spaces: present (0); absent (1) (You *et al*. 2003b, character 32). T1

81. Dentary teeth, morphology of marginal denticles: tongue-shaped with smooth edges (0); tongue-shaped with mammillated edges (1); reduced to small mammillated papillae (2). T2 [ordered]

82. Dentary teeth, number of replacement teeth per tooth position: one (0); two (1); three (2) (modified from Weishampel *et al.,* 1993, character 32). T3 [ordered]

83. Dentary teeth, number of teeth per tooth position forming part of occlusal plane: one (0); two (1); three (2) (modified from Norman 2002, character 39). T4 [ordered]

84. Dentary teeth, shape of crown in lingual view: mesiodistally broad, oblong, shield-like surface (0); mesiodistally narrow and diamond-shaped (1) (modified from Norman 2002, character 29). T5

85. Dentary teeth, ridges on lingual surface of crown: absent (0); present (1). T6

86. Dentary teeth, position of primary ridge: mesially offset (0); distally offset (1); no offset, primary ridge divides the lingual side of the crown into equal halves (2) (modified from You *et al.,* 2003, character 39). T7 [ordered]

87. Dentary teeth, number and morphology of ridges on lingual surface of crown: prominent primary ridge and multiple separate faint accessory ridges on both sides of it (0); prominent primary ridge and multiple evenly-spaced accessory ridges on either side such that entire lingual surface is corrugated (1); parallel and similarly prominent primary and secondary ridges with multiple faint accessory ridges arising from marginal denticles (2); primary ridge and a single less prominent accessory ridge on either side (3); primary ridge and a single mesial accessory ridge (4); primary ridge only (5). T8 [ordered]

88. Maxillary teeth, intercrown spaces: present (0); absent (1). T9

89. Maxillary teeth, number of teeth per tooth position forming part of occlusal plane: one (0); two (1). T10

90. Maxillary teeth, ridges on labial surface of crown: absent (0); present (1). T11

91. Maxillary teeth, primary ridge position and morphology: distally offset (0); no offset, primary ridge divides the labial side of the crown into equal halves (1) (modified from You *et al.,* 2003b, character 36). T12

92. Maxillary teeth, number and morphology of ridges on labial surface of crown: primary ridge with multiple parallel accessory ridges on either side (0); multiple ridges of similar size, such that primary ridge cannot be distinguished (1); primary ridge and only mesial accessory ridges (2); primary ridge only (3). T13 [ordered]

**Postcranial (Axial)**

93. Axis, morphology of axial neural spine in lateral view: caudodorsally sloping process (0); dorsally expanded process (1). AX1

94. Cervical vertebrae, opsisthocoely of centra: slightly opisthocoelous, flat or slightly convex rostral face (0); deeply opisthocoelous, hemispherical rostral face protrudes beyond ventral and dorsal surfaces of centrum and has a smooth, rounded surface (1). AX2

95. Ossified epaxial tendons, arrangement along dorsal, sacral, and caudal vertebrae: longitudinally arranged (0); double-layered lattice (1) (Weishampel *et al.,* 2003, character 42). AX3

**Postcranial (Appendicular)**

96. Sternal, caudolateral process: absent (0); present (1) (modified from Kobayashi and Azuma 2003, character 23). PC1

97. Sternal, caudomedial process: absent (0); present (1). PC2

98. Sternal, shape of main body in dorsal or ventral view, excluding caudolateral process if present: convex medially and concave laterally (0); convex medially and straight laterally (1). PC3

99. Scapula, dorsal margin of scapular shaft: straight (0); convex (1). PC4

100. Scapula, expansion of caudal end: gently convex expansion along caudodorsal margin, caudoventral margin tapers into hook-like flange (0); caudal end paddle-shaped, dorsal and ventral margins of scapula diverge towards caudal end (1); caudal margin of scapula straight, dorsal and ventral margins are parallel approaching caudal margin of scapula and meet caudal margin at nearly right angles (2). PC5 [ordered]

101. Scapula, shape of acromion process in lateral view: subtriangular (0); cranial margin of process is convex (1); low, rounded protuberance (2). PC6

102. Scapula, acromion process orientation: dorsally directed (0); laterally directed (1) (Norman 2002, character 44). PC7

103. Humerus, shape of deltopectoral crest: distal margin rounded and merges gradually with the lateral margin of the humeral shaft (0); distal margin angular and merges abruptly with the lateral margin of the humeral shaft (1) (modified from Weishampel *et al.,* 1993, character 37). FL1

104. Manus, digit I: present (0); absent (1) (Norman, 2002, character 51). FL2

105. Manus, ungual of digit I, shape: claw-like (0); conical (1) (modified from Norman 2002, character 52). FL3

106. Manus, arrangement of metacarpals II-IV: spreading (0); closely appressed (1) (You *et al.,* 2003b, character 49). FL4

107. Manus, unguals of digits II and III, shape: claw-like (0); flattened and hoof-like (1) (Norman 2002, character 53). FL5

108. Manus, digit III, number of phalanges: four (0); three (1) (Weishampel *et al.,* 2003, character 53). FL6

109. Ilium, preacetabular process, cranial end: rounded (0); horizontal boot (1). PV1

110. Ilium, preacetabular process, twisting along its length such that the dorsal surface of the ilium becomes the lateral surface of the cranial end of the process: absent (0); present (1) (modified from Weishampel *et al.,* 2003, character 56). PV2

111. Ilium, dorsal margin above pubic and ischial peduncles and acetabulum: straight (0); convex (1); sinuous, convex above pubic peduncle and concave above ischial peduncle (2) (modified from Weishampel *et al.,* 2003, character 55). PV3

112. Ilium, morphology of dorsal margin of postacetabular process dorsal to ischial peduncle: smooth surface (0); laterally bulging eminence dorsal to ischial peduncle, no modification of dorsal margin (1); mediolaterally thickened dorsal margin compared to dorsal margin above pubic peduncle (2); thickened and laterally-bulging everted rim along dorsal margin (3); laterally-projecting, non-pendant shelf continuous with dorsal margin of ilium (4); pendant supraacetabular process continuous with dorsal margin of ilium (5) (modified from Norman 2002, character 56). PV4 [ordered]

113. Ilium, postacetabular process, shape in lateral view: rounded with break in slope along dorsal margin, parallel dorsal and ventral margins (0); tapers to a point with break in slope along dorsal margin, forming a distinct platform for the origin of *M. iliocaudalis* (1); tapers with no break in slope along dorsal margin (2); subrectangular with no break in slope (3) (modified from Norman 2002, character 57). PV5 [ordered]

114. Ilium, postacetabular process: caudally directed (0); process curves dorsally along its entire length, such that the both the dorsal and ventral margins curve dorsally (1). PV6

115. Pubis, cranial expansion of cranial pubic process: absent, dorsal and ventral margins parallel (0); present, dorsal and ventral margins diverge distally (1) (modified from Norman 2002, character 58). PV7

116. Pubis, dorsal margin: straight (0); concave (1). PV8

117. Pubis, caudal pubic process: approximately equal in length to ischium (0); shorter than ischium (1) (Norman 2002, character 59). PV9

118. Pubis, caudal pubic process, morphology of distal end: rounded (0); tapers to a point (1). PV10

119. Ischium, morphology of shaft: curved caudally (0); curved cranially (1); straight (2) (modified from Norman 2002, character 60). PV11

120. Ischium, morphology of distal end: rounded expansion (0); cranially expanded boot (1); bluntly truncated (2) (modified from Prieto-Márquez *et al.,* 2006b, character 126). PV12

121. Femur, curvature of shaft: distal half of shaft curved caudally (0); distal half of shaft straight (1) (Norman 2002, character 62). HL1

122. Femur, groove on caudal aspect of femoral head: present (0); absent (1) (Winkler *et al.,* 1997, character 25). HL2

123. Femur, morphology of fourth trochanter: pendant (0); broad and triangular (1); curved, laterally compressed eminence (2) (Norman 2002, character 63). HL3 [ordered]

124. Femur, location of fourth trochanter: arises on proximal half of femoral shaft (0); arises at midshaft of femur (1) (Weishampel *et al.,* 2003, character 69). HL4

125. Femur, location of insertion scar of *M. caudifemoralis longus*: extends from fourth trochanter onto medial surface of femoral shaft (0); widely separated from fourth trochanter, restricted to medial surface of femoral shaft (1) (Ruiz-Omeñaca *et al.,* 2007). HL5

126. Femur, intercondylar extensor groove: absent (0); present (1) (Weishampel *et al.,* 2003, character 70). HL6

127. Femur, intercondylar extensor groove: shallow open trough with sides that diverge from each other cranially (0); deep open trough with parallel sides (1); partially enclosed by expansion of condyles (2); canal fully enclosed by fusion of lateral and medial condyles (3) (modified from Norman 2002, character 64). HL7 [ordered]

128. Femur, intercondylar flexor groove: completely open, U-shaped trough (0); partially closed by lateral inflation of medial condyle (1) (modified from Weishampel *et al.,* 2003, character 71). HL8

129. Pes, prominent lip extending proximodorsally from dorsal margins of phalangeal proximal articulation facets: present (0); absent (1). HL9

130. Pes, morphology of unguals on digits II-IV: dorsoventrally flattened, but elongate and pointed (0); dorsoventrally flattened and elongate, but with blunt truncated tips (1); hoof-like shape (2) (modified from Norman 2002, character 67). HL10 [ordered]
