# Supplementary material for: New Basal Iguanodonts from the Cedar Mountain Formation of Utah and the Evolution of Thumb-Spiked Dinosaurs
Source: PLoS One. 2010 Nov 22;5(11):e14075. doi: 10.1371/journal.pone.0014075 (PMC2989904; doi:10.1371/journal.pone.0014075)
Supplement: File S4 — List of the references cited in the character and specimen lists. (0.05 MB DOC) [file pone.0014075.s004.doc]

**References Cited in the Character List and Specimen Table (there is some overlap between this list of references and those cited in the body of the paper)**

Allain R, Pereda Suberbiola X (2003) Dinosaurs of France. Comptes Rendus Palevol 2: 27–44.

Barrett PM (1996) The first known femur of *Hylaeosaurus armatus* and re-identification of

ornithopod material in The Natural History Museum, London. Bulletin of the Natural History Museum of London (Geology) 52: 115–118.

Barrett PM, Butler RJ, Wang X-L, Xu X (2009) Cranial anatomy of the iguanodontoid

ornithopod *Jinzhousaurus yangi* from the Lower Cretaceous Yixian Formation of China. Acta Palaeontologica Polonica 54: 35–48.

Bartholomai A, Molnar RE (1981) *Muttaburrasaurus*, a new iguanodontid (Ornithischia:

Ornithopoda) dinosaur from the Lower Cretaceous of Queensland. Memoirs of the

Queensland Museum 20: 319–349.

Brill K, Carpenter K (2007) A description of a new ornithopod from the Lytle Member of

the Purgatoire Formation (Lower Cretaceous) and a reassessment of the skull of

*Camptosaurus*. In: Carpenter K, editor. Horns and Beaks: Ceratopsian and Ornithopod Dinosaurs. Bloomington: Indiana University Press. pp. 49–67.

Brown B (1914) *Corythosaurus casuarius*, a new crested dinosaur from the Belly River

Cretaceous, with provisional classification of the family Trachodontidae. Bulletin of the American Museum of Natural History 33: 559–564.

Brown B (1916) *Corythosaurus casuarius*: skeleton, musculature and epidermis. Bulletin of the

American Museum of Natural History 35: 709–716.

Carpenter K, Wilson Y (2008) A new species of *Camptosaurus* (Ornithopoda: Dinosauria)

from the Morrison Formation (Upper Jurassic) of Dinosaur National Monument, Utah,

and a biomechanical analysis of its forelimb. Annals of the Carnegie Museum 76: 227–

263.

Carpenter K, Dilkes D, Weishampel DB (1995) The dinosaurs of the Niobrara Chalk

Formation (Upper Cretaceous, Kansas). Journal of Vertebrate Paleontology 15: 275–297.

Cooper MR (1985) A revision of the ornithischian dinosaur *Kangnasaurus coetzeei* Haughton,

with a classification of the Ornithischia. Annals of the South African Museum 95: 281–

317.

Dalla Vecchia FM (2009a) *Telmatosaurus* and the other hadrosauroids of the Cretaceous

European Archipelago. An update. Natura Nascosta 39: 1–18.

Dalla Vecchia, FM (2009b) *Tethyshadros insularis*, a new hadrosauroid dinosaur (Ornithischia)

from the Upper Cretaceous of Italy. Journal of Vertebrate Paleontology 29: 1100–1116.

DiCroce T, Carpenter K (2001) New ornithopod from the Cedar Mountain Formation (Lower

Cretaceous) of eastern Utah. In: Tanke DH, Carpenter K, editors. Mesozoic Vertebrate Life. Bloomington: Indiana University Press. pp. 183–196.

Forster CA (1990) The postcranial skeleton of the ornithopod dinosaur *Tenontosaurus tilletti*.

Journal of Vertebrate Paleontology 10: 273–294.

Galton PM (1974) The ornithischian dinosaur *Hypsilophodon* from the Wealden of the Isle of

Wight. Bulletin of the British Museum (Natural History) Geology 25: 1–152.

Galton PM (1981) *Dryosaurus*, a hypsilophodontid dinosaur from the Upper Jurassic of North

America and Africa. Postcranial Skeleton. Paläontologische Zeitschrift 55: 271–312.

Galton PM (1983) The cranial anatomy of *Dryosaurus*, a hypsilophodontid dinosaur from the

Upper Jurassic of North America and East Africa, with a review of hypsilophodontids

from the Upper Jurassic of North America. Geologica et Palaeontologica 17: 207–243.

Galton PM (2009) Notes on Neocomian (Lower Cretaceous) ornithopod dinosaurs from

England - *Hypsilophodon*, *Valdosaurus*, “*Camptosaurus*”, “*Iguanodon*” - and referred specimens from Romania and elsewhere. Revue de Paléobiologie, Genève 28: 211–273.

Galton PM, Powell HP (1980) The ornithischian dinosaur *Camptosaurus prestwichii*

from the Upper Jurassic of England. Palaeontology 23: 411–443.

Galton PM, Taquet P (1982) *Valdosaurus*, a hypsilophodontid dinosaur from the Lower

Cretaceous of Europe and Africa. Geobios 15: 147–159.

Gates TA, Sampson SD (2007) A new species of *Gryposaurus* (Dinosauria: Hadrosauridae) from

the late Campanian Kaiparowits Formation, southern Utah, USA. Zoological Journal of the Linnean Society 151: 351–376.

Gilmore CW (1909) Osteology of the Jurassic reptile *Camptosaurus*, with a revision of the

species of the genus, and descriptions of two new species. Proceedings of the United

States National Museum 36: 197–332.

Gilmore CW (1933) On the dinosaurian fauna of the Iren Dabasu Formation. Bulletin of the

American Museum of Natural History 67: 23–78.

Gilpin D, DiCroce T, Carpenter K (2007) A possible new basal hadrosaur from the Lower

Cretaceous Cedar Mountain Formation of eastern Utah. In: Carpenter K, editor. Horns and Beaks: Ceratopsian and Ornithopod Dinosaurs. Bloomington: Indiana University Press. pp. 79–89.

Godefroit P, Codrea V, Weishampel DB (2009) Osteology of *Zalmoxes shqiperorum*

(Dinosauria, Ornithopoda), based on new specimens from the Upper Cretaceous of Nǎlaţ-Vad (Romania). Geodiversitas 31: 525–553.

Godefroit P, Li H, Shang C-Y (2005) A new primitive hadrosauroid dinosaur from the

Early Cretaceous of Inner Mongolia (P. R. China). Comptes Rendus Palevol 4: 697–705.

Godefroit P, Dong Z-M, Bultynck P, Li H, Feng L (1998) New *Bactrosaurus*

(Dinosauria: Hadrosauroidea) material from Iren Dabasu (Inner Mongolia, P. R. China). Bulletin de l’Institut Royal des Sciences Naturelles de Belgique, Sciences de la Terra 68 (Supplement): 3–70.

Head JJ (1998) A new species of basal hadrosaurid (Dinosauria, Ornithischia) from the

Cenomanian of Texas. Journal of Vertebrate Paleontology 18: 718–738.

Head JJ (2001) A reanalysis of the phylogenetic position of *Eolambia caroljonesa*

(Dinosauria, Iguanodontia). Journal of Vertebrate Paleontology 21: 392–396.

Hooley RW (1925) On the skeleton of *Iguanodon atherfieldensis* sp. nov., from the Wealden

Shales of Atherfield (Isle of Wight). Quarterly Journal of the Geological Society of

London 81: 1–61.

Horner JR, Weishampel DB, Forster CA (2004) Hadrosauridae. In Weishampel DB, Dodson P,

Osmólska H, editors. The Dinosauria: Second Edition. Berkeley: University of California Press. pp. 438–463.

Janensch W (1955) Der ornithopode *Dysalotosaurus* der Tendaguruschichten. Palaeontographica

Supplement 7: 105–176.

Kirkland JI (1998) A new hadrosaurid from the upper Cedar Mountain Formation (Albian-

Cenomanian: Cretaceous) of eastern Utah - the oldest known hadrosaurid (lambeosaurine?). In: Lucas SG, Kirkland JI, Estep JW, editors. Lower and Middle Cretaceous Terrestrial Ecosystems. New Mexico Museum of Natural History and Science Bulletin 14: 283–295.

Kobayashi Y, Azuma Y (2003) A new iguantodontian (Dinosauria: Ornithopoda) from the

Lower Cretaceous Kitadani Formation of Fukui Prefecture, Japan. Journal of Vertebrate Paleontology 23: 166–175.

Langston Jr. W (1960) The vertebrate fauna of the Selma Formation of Alabama, Part VI: the

dinosaurs. Fieldiana: Geology Memoirs 8: 319–360.

Lü J (1997) A new Iguanodontidae (*Probactrosaurus mazongshanensis* sp. nov.) from

Mazongshan Area, Gansu Province, China. In: Dong Z, editor. Sino-Japanese

Silk Road Dinosaur Expedition. Beijing: China Ocean Press. pp. 27–47.

Lydekker R (1888) Note on a new Wealden iguanodont and other dinosaurs. Quarterly Journal

of the Geological Society of London 44: 46–61.

Lydekker R (1889) Notes on new and other dinosaurian remains. Geological Magazine, Series

3, 6: 352–356.

Marsh OC (1893) The skull and brain of *Claosaurus*. American Journal of Science, Series 3,

45: 83–86.

Mateus O, Antunes MT (2001) *Draconyx loureiroi*, a new Camptosauridae (Dinosauria,

Ornithopoda) from the Late Jurassic of Lourinhã, Portugal. Annales de Paléontologie 87: 61–73.

McDonald AT, Barrett PM, Chapman, SD (2010a) A new basal iguanodont (Dinosauria:

Ornithischia) from the Wealden (Lower Cretaceous) of England. Zootaxa 2569: 1–43.

McDonald AT, Wolfe DG, Kirkland JI (2010b) A new basal hadrosauroid (Dinosauria:

Ornithopoda) from the Turonian of New Mexico. Journal of Vertebrate Paleontology 30: 799–812.

Molnar RE (1996) Observations on the Australian ornithopod dinosaur, *Muttaburrasaurus*.

Memoirs of the Queensland Museum 39: 639–652.

Norman DB (1980) On the ornithischian dinosaur *Iguanodon bernissartensis* from Belgium.

Mémoires Institut Royal des Sciences Naturelles de Belgique 178: 1–103.

Norman DB (1986) On the anatomy of *Iguanodon atherfieldensis* (Ornithischia: Ornithopoda).

Bulletin de L’Institut Royal des Sciences Naturelles de Belgique, Sciences de la Terre 56: 281–372.

Norman DB (1998) On Asian ornithopods (Dinosauria: Ornithischia). 3. A new species of

iguanodontid dinosaur. Zoological Journal of the Linnean Society 122: 291–348.

Norman DB (2002) On Asian ornithopods (Dinosauria: Ornithischia). 4. *Probactrosaurus*

Rozhdestvensky, 1966. Zoological Journal of the Linnean Society 136: 113–144.

Norman DB (2004) Basal Iguanodontia. In: Weishampel DB, Dodson P, and Osmolska H,

editors. The Dinosauria: Second Edition. Berkeley: University of California Press. pp. 413–437.

Norman DB (2010) A taxonomy of iguanodontians (Dinosauria: Ornithopoda) from the lower

Wealden Group (Cretaceous : Valanginian) of southern England. Zootaxa 2489: 47–66.

Norman DB, Barrett PM (2002) Ornithischian dinosaurs from the Lower Cretaceous

(Berriasian) of England. Special Papers in Palaeontology 68: 161–189.

Ostrom JH (1961) Cranial morphology of the hadrosaurian dinosaurs of North America. Bulletin

of the American Museum of Natural History 122: 33–186.

Ostrom JH (1970) Stratigraphy and paleontology of the Cloverly Formation (Lower Cretaceous)

of the Bighorn Basin Area, Wyoming and Montana. Peabody Museum Bulletin 35: 1–233.

Prieto-Márquez A (2010) Global phylogeny of hadrosauridae (Dinosauria: Ornithopoda) using

parsimony and Bayesian methods. Zoological Journal of the Linnean Society 159: 435–502.

Prieto-Márquez A, Weishampel DB, Horner JR (2006a) The dinosaur *Hadrosaurus foulkii*, from

the Campanian of the East Coast of North America, with a reevaluation of the genus. Acta Palaeontologica Polonica 51: 77–98.

Prieto-Márquez A, Gaete R, Rivas G, Galobart À, Boada M (2006b) Hadrosauroid

dinosaurs from the Late Cretaceous of Spain: *Pararhabdodon isonensis* revisited and *Koutalisaurus kohlerorum*, gen. et sp. nov. Journal of Vertebrate Paleontology 26: 929–943.

Ruiz-Omeñaca JI, Pereda Suberbiola X, Galton PM (2007) *Callovosaurus leedsi*, the

earliest dryosaurid dinosaur (Ornithischia: Euornithopoda) from the Middle Jurassic of

England. In: Carpenter K, editor. Horns and Beaks: Ceratopsian and Ornithopod Dinosaurs. Bloomington: Indiana University Press. pp. 3–16.

Sereno PC (1991) *Lesothosaurus*, “fabrosaurids,” and the early evolution of Ornithischia.

Journal of Vertebrate Paleontology 11: 168–197.

Sues H-D, Averianov A (2009) A new basal hadrosauroid dinosaur from the Late Cretaceous of

Uzbekistan and the early radiation of duck–billed dinosaurs. Proceedings of the Royal Society B 276: 2549–2555.

Taquet P (1976) Ostéologie d’*Ouranosaurus nigeriensis*, Iguanodontide du Crétacé Inférieur du

Niger. Géologie et Paléontologie du Gisement de Gadoufaoua (Aptien du Niger), Chapitre III. pp. 57–168.

Taquet P, Russell DA (1999) A massively-constructed iguanodont from Gadoufaoua, Lower

Cretaceous of Niger. Annales de Paléontologie 85: 85–96.

Weishampel DB, Bjork PR (1989) The first indisputable remains of *Iguanodon* (Ornithischia:

Ornithopoda) from North America: *Iguanodon lakotaensis*, sp. nov. Journal of Vertebrate Paleontology 9: 56–66.

Weishampel DB, Norman DB, Grigorescu D (1993) *Telmatosaurus transsylvanicus*

from the Late Cretaceous of Romania: the most basal hadrosaurid dinosaur.

Palaeontology 36: 361–385.

Weishampel DB, Jianu C-M, Csiki Z, Norman DB (2003) Osteology and phylogeny of

*Zalmoxes* (n.g.), an unusual euornithopod dinosaur from the latest Cretaceous of

Romania. Journal of Systematic Palaeontology 1: 65–123.

Wiman C (1929) Die Kreide-dinosaurier aus Shantung. Palaeontologia Sinica 6: 1–63.

Winkler DA, Murry PA, Jacobs LL (1997) A new species of *Tenontosaurus* (Dinosauria:

Ornithopoda) from the Early Cretaceous of Texas. Journal of Vertebrate Paleontology 17: 330–348.

Xu X, Zhao XJ, Lu J-C, Huang W-B, Li Z-Y, Dong Z-M (2000) A new iguanodontian

from Sangping Formation of Neixiang, Henan and its stratigraphical implications.

Vertebrata PalAsiatica 38: 176–191.

You H-L, Li D-Q (2009) A new basal hadrosauriform dinosaur (Ornithischia: Iguanodontia)

from the Early Cretaceous of northwestern China. Canadian Journal of Earth Sciences 46: 949–957.

You H, Ji Q, Li D (2005) *Lanzhousaurus magnidens* gen. et sp. nov. from Gansu Province,

China: the largest-toothed herbivorous dinosaur in the world. Geological Bulletin of

China 24: 785–794.

You H, Ji Q, Li J, Li Y (2003a) A new hadrosauroid dinosaur from the mid-Cretaceous of

Liaoning, China. Acta Geologica Sinica 77: 148–154.

You H-L, Luo Z-X, Shubin NH, Witmer LM, Tang Z-L, Tang F (2003b) The

earliest-known duck-billed dinosaur from deposits of late Early Cretaceous age in

northwest China and hadrosaur evolution. Cretaceous Research 24: 347–355.
